# Comprehensive Identification of Long Non-coding RNAs in Purified Cell Types from the Brain Reveals Functional LncRNA in OPC Fate Determination

**DOI:** 10.1371/journal.pgen.1005669

**Published:** 2015-12-18

**Authors:** Xiaomin Dong, Kenian Chen, Raquel Cuevas-Diaz Duran, Yanan You, Steven A. Sloan, Ye Zhang, Shan Zong, Qilin Cao, Ben A. Barres, Jia Qian Wu

**Affiliations:** 1 The Vivian L. Smith Department of Neurosurgery, The University of Texas Health Science Center at Houston Medical School, Houston, Texas, United States of America; 2 Center for Stem Cell and Regenerative Medicine, The Brown Foundation Institute of Molecular Medicine for the Prevention of Human Diseases, Houston, Texas, United States of America; 3 Department of Neurobiology, Stanford University School of Medicine, Stanford, California, United States of America; The Scripps Research Institute, UNITED STATES

## Abstract

Long non-coding RNAs (lncRNAs) (> 200 bp) play crucial roles in transcriptional regulation during numerous biological processes. However, it is challenging to comprehensively identify lncRNAs, because they are often expressed at low levels and with more cell-type specificity than are protein-coding genes. In the present study, we performed *ab initio* transcriptome reconstruction using eight purified cell populations from mouse cortex and detected more than 5000 lncRNAs. Predicting the functions of lncRNAs using cell-type specific data revealed their potential functional roles in Central Nervous System (CNS) development. We performed motif searches in ENCODE DNase I digital footprint data and Mouse ENCODE promoters to infer transcription factor (TF) occupancy. By integrating TF binding and cell-type specific transcriptomic data, we constructed a novel framework that is useful for systematically identifying lncRNAs that are potentially essential for brain cell fate determination. Based on this integrative analysis, we identified lncRNAs that are regulated during Oligodendrocyte Precursor Cell (OPC) differentiation from Neural Stem Cells (NSCs) and that are likely to be involved in oligodendrogenesis. The top candidate, *lnc-OPC*, shows highly specific expression in OPCs and remarkable sequence conservation among placental mammals. Interestingly, *lnc-OPC* is significantly up-regulated in glial progenitors from experimental autoimmune encephalomyelitis (EAE) mouse models compared to wild-type mice. OLIG2-binding sites in the upstream regulatory region of *lnc-OPC* were identified by ChIP (chromatin immunoprecipitation)-Sequencing and validated by luciferase assays. Loss-of-function experiments confirmed that *lnc-OPC* plays a functional role in OPC genesis. Overall, our results substantiated the role of lncRNA in OPC fate determination and provided an unprecedented data source for future functional investigations in CNS cell types. We present our datasets and analysis results *via* the interactive genome browser at our laboratory website that is freely accessible to the research community. This is the first lncRNA expression database of collective populations of glia, vascular cells, and neurons. We anticipate that these studies will advance the knowledge of this major class of non-coding genes and their potential roles in neurological development and diseases.

## Author Summary

Between 70 and 90% of the mammalian genome is transcribed at some point during development; however, only < 2% of the genome is associated with protein-coding genes. Emerging evidence suggests that long non-coding RNAs (lncRNAs; > 200 bp) play important roles in cell fate determination. In the present study, we broadened the lncRNA catalog by *ab initio* reconstruction of the transcriptomes of purified mouse cortex cell populations. More than 5000 lncRNAs were detected in the brain cell types studied. Predicting lncRNA functions using a ‘guilt-by-association’ approach revealed potential functions of lncRNAs in Central Nervous System development. Additionally, we analyzed transcription factor occupancy in the upstream regulatory regions of the lncRNAs. By integrating differential gene expression and transcription factor occupancy information, lncRNAs that are likely involved in oligodendrocyte precursor cell formation were identified. Loss-of-function experiments confirmed that the top candidate, *lnc-OPC* (long non-coding RNA in OPC), significantly reduces OPC differentiation from NSCs. Interestingly, *lnc-OPC* is up-regulated in glial progenitors of mouse models for multiple sclerosis. Our results demonstrated the role of lncRNA in the context of oligodendrocyte cell fate determination, and provided an extensive resource and a powerful analysis framework for future functional investigations of lncRNAs in CNS cell types.

## Introduction

More than 98% of the human genome does not encode proteins. A large number of transcribed sequences are non-coding transcripts [1–5]. Thousands of long non-coding RNAs (lncRNAs: usually > 200 bp in length, often spliced and polyadenylated, but lacking protein-coding potential) were recently discovered and many of them have been shown to play crucial roles in diverse biological processes [6, 7]. Emerging evidence indicates that lncRNAs may have important roles in Central Nervous System (CNS) development, homeostasis, stress responses, and plasticity [6]. For example, many lncRNAs are expressed in the mouse brain and show region-specific expression patterns [8]. Many lncRNAs exhibit dynamic expression patterns during neuronal-glial fate specification and oligodendrocyte lineage maturation [6]. In addition, lncRNAs have been shown to be involved in some neuropsychiatric diseases [9].

An increasing effort is being devoted to lncRNA identification [2, 3, 10]; however, it is not trivial to build a comprehensive lncRNA catalog. Compared to their protein-coding counterparts, lncRNAs are generally expressed at lower levels, which make it difficult to detect and assemble these transcripts, especially if the lncRNAs are expressed in the minor cell types within a tissue [2, 3, 8]. In addition, lncRNA genes may be regulated in opposing directions in different cell types, so their expression can appear to be static in composite tissue data. Traditionally, microarrays were used to capture lncRNA, but microarray data is limited in its sensitivity and by the probes that can match lncRNAs. Targeted capture (using tiling arrays to target selected portions of the transcriptome) followed by RNA-Sequencing (RNA-Seq) can be used to validate transcripts that are expressed at low levels [10, 11]; however, this approach requires *a priori* knowledge of the target region. Another challenge is to investigate the potential functions of lncRNAs. The functional roles of most characterized lncRNAs were first inferred by transcriptional profiling of different samples, an approach that presumes a cause-and-effect relationship between gene expression and cellular context. This ‘guilt-by-association’ strategy has proven to be a powerful tool for discovering the biological functions of lncRNAs [2, 12]. Nevertheless, it is still critically important to validate the predicted functions of lncRNAs by classical genetic approaches such as loss-of-function experiments [13]. Additional genomic information, such as specific transcription factor binding that provides evidence of active regulation, can increase the precision of candidate selection for functional validation experiments [14].

We have recently employed RNA-Seq to characterize the transcriptome of various purified cell types isolated from mouse brain [15]. The expression levels of classic cell-type specific markers were high in their corresponding cell types, but very low in the other cell populations, demonstrating the high purity of the isolated brain cell types (S1 Table; pericytes were excluded from the analyses because of relatively lower purity). In the current study, we also sequenced neural stem cells. We identified lncRNAs *de novo* from these purified cell types and generated a more comprehensive lncRNA annotation database by combining the lncRNAs that we identified with those from multiple other sources including GENCODE, RefSeq, Ensembl, lncRNAdb, and lncRNAs recently identified by several other groups [3, 4, 10, 16, 17]. Predicting lncRNA functions using purified cell types revealed potential functions for lncRNAs in CNS development. Moreover, to further dissect the functional roles of these lncRNAs, we performed TF motif searches in ENCODE DNase I digital footprint (DNase-DGF) experimental data and Mouse ENCODE promoters to infer TF binding proximal to these lncRNAs at various CNS developmental stages [18].

This comprehensive database of lncRNAs from purified brain cell types that we have integrated with TF binding and predicted functional information provides a powerful framework for systematically identifying lncRNAs that are essential for brain cell fate determination. Because Oligodendrocyte Precursor Cells (OPCs) play a crucial role in myelination/remyelination, and understanding the determination of OPC fate is critical for harnessing their potential for cell-based therapies [19], we chose to investigate lncRNAs that may have essential functions in OPC fate determination. Based on our integrative analysis, the top candidate, which we named *lnc-OPC*, showed highly specific expression in OPCs, remarkable sequence conservation among placental mammals, and OLIG2 binding in its upstream regulatory region, as shown by ChIP-Seq and luciferase assays. Furthermore, the depletion of *lnc-OPC* significantly reduces OPC formation and affects global expression of genes associated with oligodendrogenesis upon the differentiation of OPCs from NSCs. Interestingly, we found transposable elements (TE) inserted in the intron of *lnc-OPC* in the mouse lineage, implying that TEs might have been involved in the evolution and regulation of the expression of *lnc-OPC*.

These results substantiated the role of lncRNA in OPC determination and established a valuable framework that can be applied to future large-scale functional lncRNA screens in other cell types. We have presented our datasets and analytical results as online resources freely available to the research community (http://jiaqianwulab.org/braincell/lncRNA.html) (Username: lncRNA; Password: rnaseq). We anticipate that our study will advance the knowledge of this major class of non-coding genes and their potential roles in neurological development and diseases.

## Results

### A broadened lncRNA catalog from brain cell-type specific RNA-Seq data

**Fig 1:**
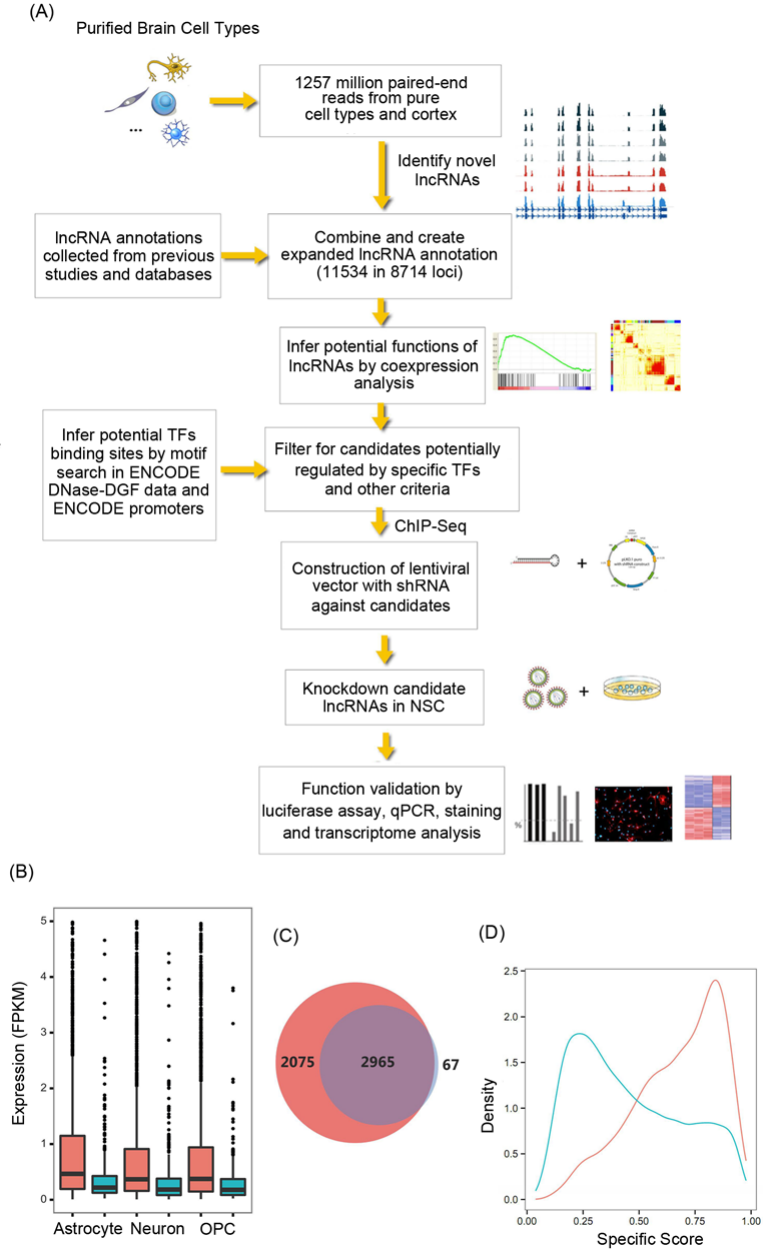
Overview of study diagram and evaluation of lncRNA expression. (A) Schematic of the data integration and experiment validation. (B) Box plots illustrate expression level distributions of lncRNAs detected in Astrocytes, Neurons, and OPC cells (Red: lncRNAs that are also detected in cortex tissue samples; Blue: lncRNAs that are not detected in cortex tissue samples. Any lncRNAs with expression level of FPKM > 5 were excluded to allow the plot to be presented at a suitable scale. (C) Venn diagram shows lncRNAs detected in purified cell samples (red) or tissue samples (blue). (D) Cell-type specificity of the expression patterns of lncRNAs. Shown are the distributions (represented as a density curve) of specificity scores calculated for each gene across cell types, for coding genes (blue) and lncRNAs (red). The specificity score was calculated with a previously proposed index, which varies from 0 for housekeeping genes to 1 for cell-type specific genes.

Previous mouse brain lncRNA catalogs created using tissue or organ samples might have missed lncRNAs that can only be found in minor cell types and are hence likely not comprehensive [10]. To further broaden current lncRNA catalogs, we set out to identify lncRNAs that are expressed in various purified brain cell types by employing an *ab initio* transcriptome reconstruction approach. We previously reported RNA sequencing of poly(A)+ mRNA from mouse cerebral cortex tissue samples, as well as from highly purified astrocytes, neurons, oligodendrocyte precursor cells (OPCs), newly formed oligodendrocytes (NFOs), myelinating oligodendrocytes (MOs), microglia (MGL), endothelial cells (Endo), and pericytes (Peri) [15]. In this previous work, we reported 811 lncRNAs with the criterion of FPKM > 1 from the eight brain cell types based on GENCODE annotation. In order to better study transcriptome dynamics during cell lineage commitment, we now include the published RNA-Seq data for mouse embryonic stem cells (ESCs) [3] in our analyses. In addition, we have also sequenced transcripts from primary NSC isolated from mouse cortex using the same library construction procedures as described previously [15, 20]. In total, ~1.2 billion 101-bp paired-end reads were collected from nine cell types and from samples of the whole cortex (average ~63 million reads/sample) (Fig 1A). We performed *ab initio* transcript assembly and detected expression of 5040 (5107, including the whole cortex samples) multi-exonic lncRNAs from 4059 loci in these brain cell types. There are 1717 novel lncRNA loci compared to the IncRNA genes annotated by GENCODE (M3 version) and RefSeq. *Ab initio* transcriptome reconstruction using cortex samples alone (at the same sequencing depth) recovered only 3032 lncRNAs (from 2519 loci), among which 1080 loci are novel compared to GENCODE and RefSeq. Most lncRNAs not detected in cortex samples were expressed at lower levels in respective cell types (Fig 1B and 1C), indicating the limitation of the data obtained from tissues and highlighting the importance of cell-type specific transcriptome profiling for lncRNA identification. Using a previously proposed index [21], we evaluated the cell specificity of the expression patterns of lncRNAs, as well as that of protein-coding genes. Consistent with previous observations, the lncRNA genes showed more cell-type specific expression patterns than protein-coding genes (Fig 1D).

In an effort to obtain a comprehensive lncRNA catalog for downstream analysis, we surveyed available lncRNA annotations in the public domain. Numerous lncRNA annotations were retrieved from databases including lncRNAdb, GENCODE, Ensembl, and UCSC known gene and RefSeq genes. Additionally, we also incorporated lncRNA annotations from several recent transcriptome studies performed using RNA-Seq technology (see Materials and Methods) [3, 4, 10]. Because the definition of a full-length transcript boundary is not always accurate without further experimental evidence, we merged lncRNA transcripts identified from the same loci that were not annotated by GENCODE, or by any other lncRNA databases, into single transcripts. This merging procedure reduced the apparent total number of lncRNA transcripts, but increased the accuracy of expression level estimation, which is more important for prediction of function [22]. Thus, our final lncRNA annotation includes a total of 11,534 lncRNA transcripts from 8714 loci. Finally, we combined the comprehensive lncRNA annotation with all known UCSC genes (from the iGenome package) and built a non-redundant annotation. We quantified the expression levels of all annotated transcripts across cell types using this combined annotation (S2 Table).

### Predicting potential functions of lncRNAs differentially expressed in brain cell types

**Fig 2:**
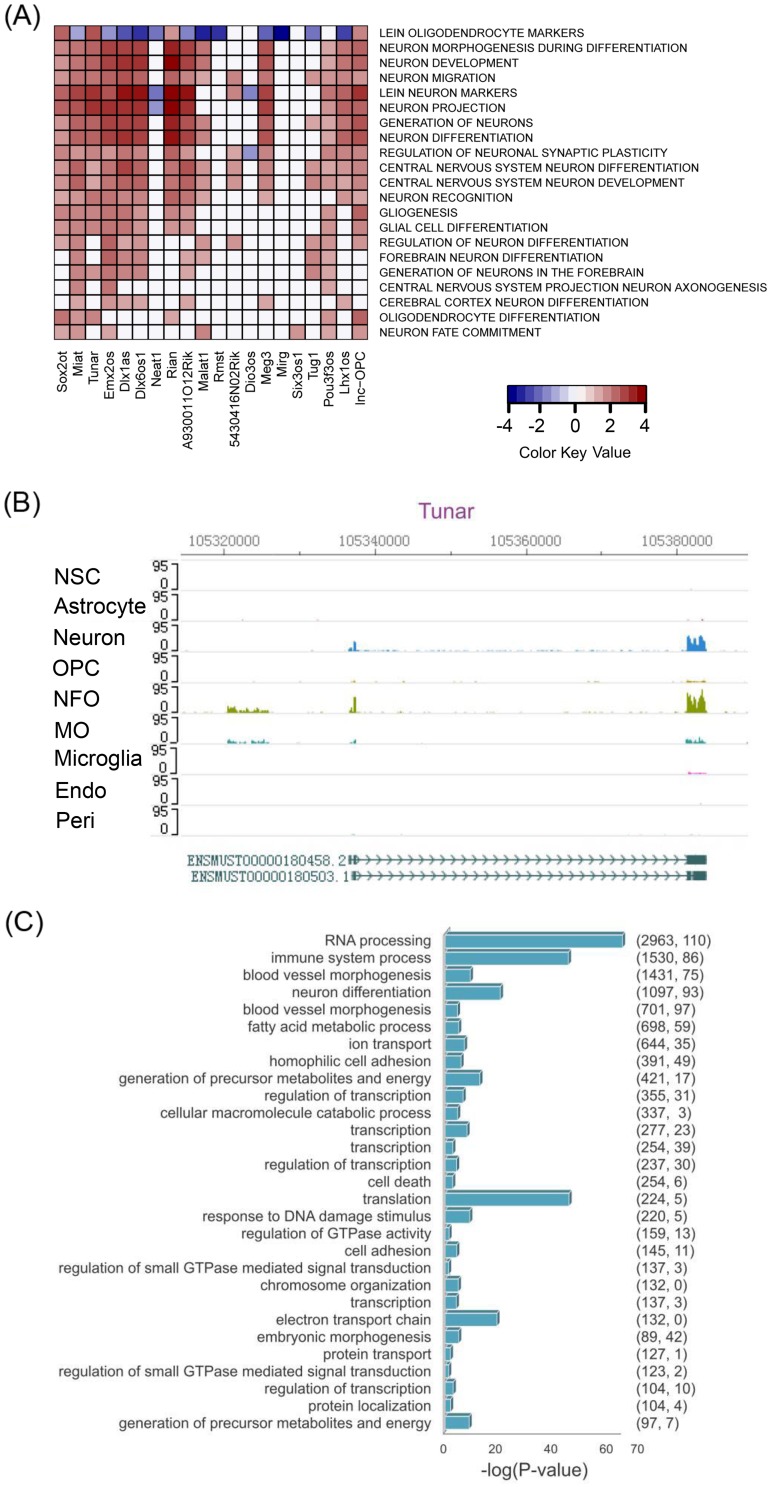
Predicting putative functions of lncRNAs. (A) Shown is a heatmap representing an association matrix of lncRNAs and functional terms. Columns represent lnc-OPC and lncRNAs that are known to be expressed in brain and have known functions in the literature. Rows represent selected gene ontology terms and MsigDB gene sets. Color depth represents NES (normalized enrichment score) calculated by GSEA, indicating the strength of association. (B) RNA-Seq signal tracks for lncRNA *Tunar* across cell types are shown. All tracks are set to the same scale for easy comparison of expression levels. (C) GO enrichment analysis of co-expression modules identified by WGCNA. Only the most significant GO Biological Process terms are displayed for modules with > 100 members.

Recent efforts in lncRNA functional prediction using publicly available microarray or RNA-Seq data from tissue samples are limited due to the data source [4, 23–25]. In order to infer the potential functions of lncRNAs involved in CNS development, we adopted a previously proposed ‘guilt-by-association’ approach [4]. We used RNA-Seq profiles from fifteen types of samples (eight brain cell types and Mouse ENCODE RNA-Seq data of seven non-brain tissues including thymus, testis, kidney, liver, lung, spleen, and heart (GEO accession GSE36025)). The protein-coding genes were ranked by the Pearson correlation of their expression profile with that of a particular lncRNA. The ranked list of these protein-coding genes was then used for Gene Set Enrichment Analysis (GSEA) to identify significantly enriched gene sets. This procedure was performed for all lncRNAs against gene sets generated from Gene Ontology (GO) functional terms, canonical pathways, and expert-curated gene sets [26]. We then created an association matrix between functional terms and lncRNAs (S3 Table). Only enriched gene sets, with false discovery rates (FDR) < 0.25 (as recommended in the GSEA manual), were used for matrix creation. To validate the reliability of predicted functions, we examined *lnc-OPC* and lncRNAs that are known to be expressed in brain and have known functions in the literature [16] (Fig 2A). The predicted functions of these lncRNAs matched those in the literature well, and revealed additional associated functions. For example, lncRNA *Malat1* (metastasis-associated lung adenocarcinoma transcript 1) is a highly abundant nucleus-restricted RNA that localizes to nuclear speckles and was suggested to coordinate the RNA polymerase II transcription, pre-mRNA splicing, and mRNA export [27]. Recent studies have also shown that *Malat1* is involved in cell-cycle progression and that it enhances cellular proliferation [28]. Consistent with that result, our functional prediction for *Malat1* is closely associated with terms such as ‘Pediatric cancer markers’, ‘Establishment of RNA localization’, ‘RNA transport’, ‘Cell division’, ‘Mitotic cell cycle’, and ‘Cell cycle phase’, among others. Interestingly, other functional terms, such as ‘Forebrain development’, ‘Neural tube development’, and ‘Brain development’ were also significantly associated with *Malat1*. Furthermore, we selected a recently characterized functional lncRNA, *Tuna* (also named *Tunar*), as an example to illustrate the importance of using cell-type specific data for function prediction. The lncRNA *Tuna* is evolutionarily conserved and displays CNS-specific expression patterns [13]. Loss-of-function experiments have revealed that *Tuna* is required for neuronal differentiation, and depletion of *Tuna* in zebrafish greatly impaired locomotor functions. The authors thus concluded that depletion of *Tuna* impairs CNS function, probably due to neuronal defects. Consistent with this observation, in our analysis, gene sets such as ‘neuron markers’, ‘synapse’, ‘transmission of nerve impulse’, and ‘neuron projection’ are among the most highly enriched terms for *Tuna*. Intriguingly, the gene set ‘oligodendrocyte markers’ is also highly enriched. We then checked the expression level of *Tuna* across all brain cell types and found that the expression of *Tuna* in newly formed oligodendrocytes is comparable to that in neurons (NFO: FPKM = 5.78; Neuron: FPKM = 3.77) (Fig 2B). Thus, *Tuna* might also have a functional role in oligodendrocytes, which could be one reason that inhibiting *Tuna* causes impaired locomotor function. Such observations could not be made if cell-type specific transcriptomic data were not available.

We also performed weighted gene co-expression network analysis (WGCNA) and constructed co-expression networks comprised of both protein-coding and lncRNA genes using cell-type specific RNA-Seq data (S1 Fig). We identified 32 co-expression modules. For 29 co-expression modules that have >100 members, we performed functional term enrichment analysis using the DAVID bioinformatics tools (Figs 2C and S2 Fig; S4 and S5 Tables). Interestingly, three modules were associated with Alzheimer’s disease, Huntington’s disease and Parkinson’s disease. A close look at these modules revealed that they are all enriched for mitochondrion-related functional terms. Consistently, a number of studies have suggested that mitochondria could play a critical role in neurodegenerative diseases [29, 30]. This raises the possibility that the 24 lncRNAs found in these modules may have potential roles in regulating mitochondrial gene expression and may be related to neurodegenerative diseases.

### lncRNAs are actively regulated by TFs during CNS development

The lower expression levels of lncRNAs relative to their protein-coding counterparts raised the question of whether lncRNAs are actively regulated, or whether their expression is merely transcriptional noise [31]. To address questions as to the involvement of lncRNAs in CNS development and to facilitate functional tests of these possible roles, we analyzed the binding status of transcription factor binding sites across the whole genome. In eukaryotes, transcription is regulated in a cell-type and condition-specific manner through the association of transcription factors with the chromatin. The dynamics of chromatin accessibility in the regulatory regions of lncRNAs during developmental processes can be used as a further indicator of active regulation. We used genome-wide maps of *in vivo* DNase I footprints data retrieved from ENCODE to assess the dynamics of transcription factor binding in the proximal regulatory regions of lncRNAs and protein-coding genes. DNase I hypersensitivity mapping and genomic footprinting have been used extensively to delineate *cis*-regulatory DNA and TF binding at nucleotide resolution in various model organisms [32, 33]. Global mapping of TF footprints provides a powerful tool for assessing the interactions of hundreds of TFs with chromatin in a single experiment. To assess the dynamics of TFs during CNS development, we analyzed genomic DNase-DGF data from ESC and three other available ENCODE datasets related to CNS development (Whole Brain E14.5 [WBE14], Whole Brain adult 8 weeks [WB8wks], and Retina Newborn 1 Day [Retina1D]). In brief, we scanned the whole mouse genome for TF-binding sites using well-annotated TF-binding motifs collected from databases and the literature, then inferred their binding status from their specific DNase I cleavage profiles using a Bayesian method named CENTIPEDE [34]. Only TF-binding sites with a posterior probability of >0.99 were considered to be actively bound by TFs (S3 Fig).

**Fig 3:**
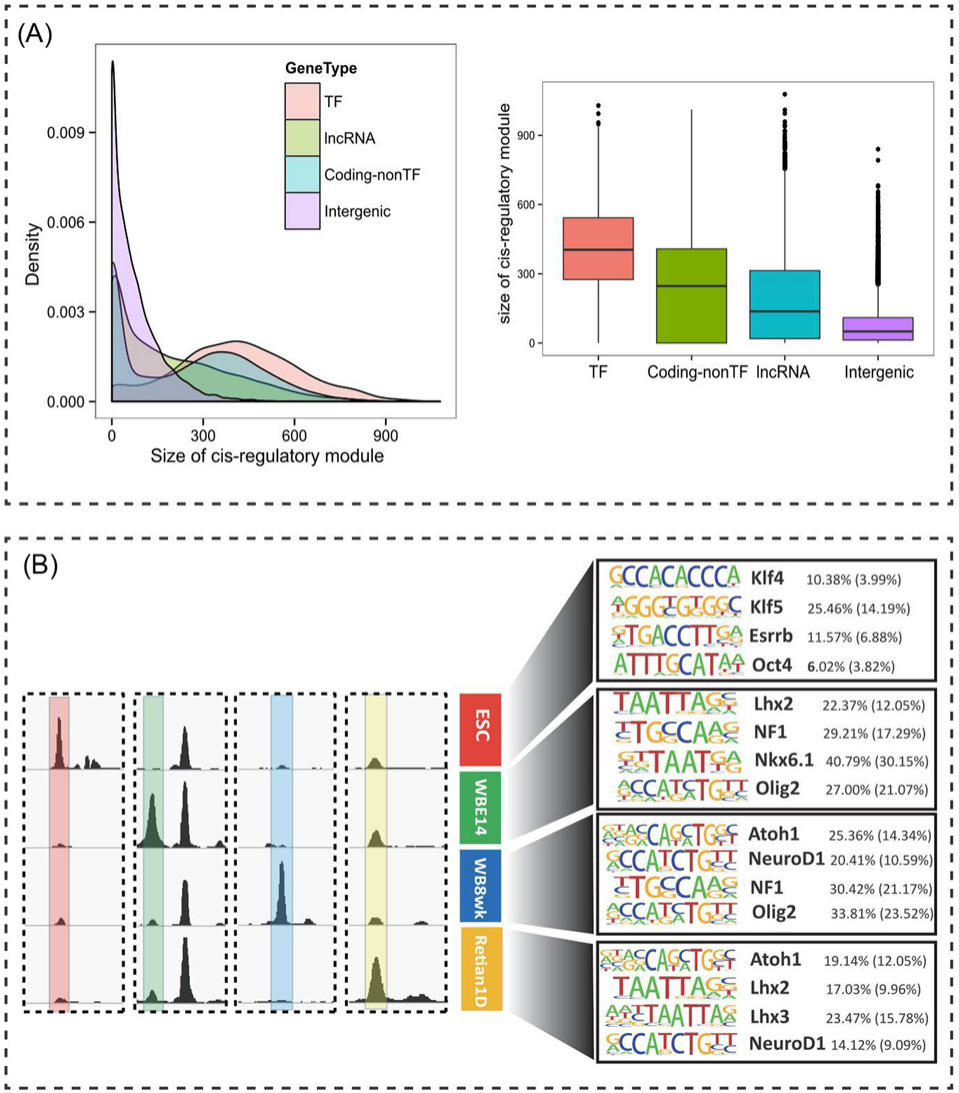
Developmental regulation of lncRNA expression by TFs. (A) (Left) The distribution of *cis*-regulatory module size in promoter regions (2 kb upstream and 1 kb downstream of TSS) of TF genes, lncRNAs, coding non-TF genes, and random intergenic regions. (Right) The distribution is illustrated with box plots. (B) (Left) Representative examples of dynamic DHSs enriched in ESC and three other available ENCODE datasets related to CNS development. (Right) Sequence motifs associated with dynamic DHSs enriched in each dataset. Top enriched representative TF motifs from *de novo* motif analysis are shown. The proportion of *cis*-regulatory sequences within dynamic DHS regions containing at least one instance of each motif is shown to the right of the motif, which is significantly enriched compared to the expected random background frequency of the motif (shown in parentheses).

Genes under complex transcriptional regulation have been suggested to be subject to a larger ‘control set’ of *cis*-regulatory modules (defined as a stretch of DNA in which a number of TFs can bind and regulate expression of nearby genes) than are genes responsive to only a few regulatory signals [35]. Using the size of *cis*-regulatory modules as an indicator, we set out to test whether lncRNAs are under the control of TFs during CNS development. To this end, we calculated the number of base pairs that are bound in the promoter regions (defined as 2 kb upstream and 1 kb downstream of the TSS) of TFs, non-TF protein-coding genes, lncRNAs, and randomly selected intergenic regions across the DNase-DGF datasets. The results of this analysis showed that, in general, TFs have larger *cis*-regulatory modules than do other categories of genes, an observation that is consistent with their role as master regulators. Importantly, we also found that lncRNA promoter regions were actively bound by transcription factors more frequently than random intergenic regions (Fig 3A). Most lncRNA genes have a smaller *cis*-regulatory module than non-TF protein-coding genes and TF genes; however, there are lncRNA genes that are part of a comparable, or even larger, *cis*-regulatory module than are protein-coding genes, suggesting a finer degree of control of their expression by TFs.

To analyze the regulatory DNA dynamics of lncRNAs, we analyzed the DNase-DGF experiment data to identify dynamic DNase I hypersensitive sites (△DHSs) at which TFs can potentially bind across the four datasets (Fig 3B, left). A large number (13,369) of △DHSs (defined as enriched more than three fold in one sample) were found to reside in proximity to lncRNA genes (within 10 kb of the TSS). There are 6157 lncRNAs that contain at least one △DHS proximal to their TSS. To examine whether the △DHSs can reflect specific TF binding in a developmental context, we first analyzed △DHSs that are specifically activated in ESC compared to three other available ENCODE datasets related to CNS development. We performed enrichment analysis of TF recognition sequences within ESC-activated △DHSs. This analysis yielded several well-known key transcription factors associated with embryonic development and pluripotency, such as KLF4/5, OCT4, SOX2, MYC, and ESRRB, among others (Fig 3B, right). This observation is consistent with previous reports showing that many lncRNAs are targets of key pluripotent transcription factors [4, 14]. We then performed enrichment analysis for TF recognition sequences in WBE14-, WB8wks-, and Retina1D-activated △DHSs in proximity to lncRNAs. Similarly, many TFs associated with CNS development were enriched, as we expected. For example, NF1, LHX2/3, NKX6.1, ISL1, and a number of basic-helix-loop-helix (bHLH) TFs, including ATOH1, OLIG2, and NEUROD1, were among the top enriched TFs (Fig 3B, right).

Taken together, our results indicate that the expression of a substantial portion of lncRNAs is under elaborate and dynamic control by TFs during CNS development, which suggests that lncRNAs do not represent mere transcriptional noise, but may indeed play various functional roles. The information regarding TF binding can also be used as a criterion for screening lncRNA candidates for experimental validation.

### Global identification of lncRNAs regulated during OPC formation

OPCs are distributed throughout the CNS and play the crucial role of differentiating into oligodendrocytes that ensheath axons with myelin during CNS development and remyelinate axons after damage [36]. Abnormal development or maintenance of myelin sheaths can impair efficient propagation of action potentials along axons, and lead to disorders such as multiple sclerosis (MS) and leukodystrophies [37, 38]. Previous studies in animal models showed that transplanted OPCs, but not mature oligodendrocytes, can myelinate; hence, OPCs can serve as a promising cell source for transplantation therapies in demyelinating diseases [36]. Thus, understanding the sophisticated molecular mechanisms of OPC fate determination is critical for harnessing OPCs for cell-based therapies.

**Fig 4:**
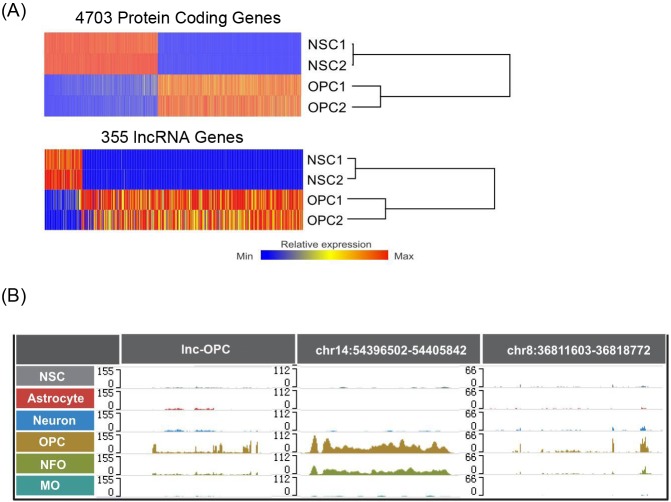
Global identification of lncRNAs regulated during oligodendrogenesis. (A) Heatmap depicting protein-coding genes and lncRNAs that are significantly differentially expressed (Fold change > 2 and FDR < 0.05) between NSCs and OPCs. Two biological replicates are represented in the plot. (B) Example RNA-Seq tracks for three lncRNAs that are enriched in the OPC lineage.

We thus set out to identify lncRNAs that could be essential for OPC fate determination. To this end, we analyzed RNA-Seq data from NSCs and OPCs. Through analysis of differential expression, we identified 4703 (Up-regulated: 2626, Down-regulated: 2077) coding genes and 355 (Up-regulated: 254, Down-regulated: 101) lncRNAs with greater than two fold (FDR < 0.05) changes in expression (Fig 4A). RNA-seq tracks for three example lncRNAs that are enriched in an OPC lineage are shown in Fig 4B. As expected, up-regulated protein-coding genes were significantly enriched for GO terms associated with oligodendrogenesis, such as ‘axon ensheathment’, ‘ensheathment of neurons’, and ‘myelination’, whereas down-regulated genes were enriched for terms associated with cell proliferation, such as ‘cell cycle’ and ‘cell division’.

Recent studies have identified non-coding RNAs that are directly regulated by key transcription factors with determinative roles in cellular differentiation [4, 14]. Based on these observations and our TF binding analysis described above, we investigated whether the differentially expressed lncRNAs are controlled by transcription factors known to play a critical role in oligodendrogenesis. During development, the bHLH TF OLIG2 is both necessary, and in some contexts, sufficient for OPC generation [39–41]. A number of studies have suggested that OLIG2 can interact with multiple TFs to provide positional cues and thus locally regulate OPC specification [42, 43]. ASCL1 (also known as Mash1) is another bHLH TF that plays an important role in OPC specification and promotes oligodendrogenesis in brain [44]. Other TFs belonging to the Homeobox and Sox family, including NKX2.2 and SOX10, also participate in oligodendrogenesis [41, 45].

We examined whether the lncRNAs that are up-regulated during oligodendrogenesis could possibly be controlled by known oligodendrogenic TFs, particularly OLIG2 and ASCL1. To this end, we analyzed previously identified genome-wide binding site locations of OLIG2 and ASCL1 in DNAse-DGF data (S6 Table). Binding sites for OLIG2 were found in proximity (within 10 kb of the TSS) to 88 (34%) of 254 lncRNAs that are up-regulated during oligodendrogenesis. Also, binding sites for ASCL1 were found close to the TSS of 111 (43%) up-regulated lncRNAs. Similarly, up-regulated protein-coding genes were enriched for OLIG2 (917 (35%)) and ASCL1 binding motifs (1246 (47%)). The enrichment of OLIG2- and ASCL1-binding sites is statistically significant, as determined by permutation analysis (p-value < 0.01, using randomly selected genes as control).

In addition to DNase-DGF analysis, we also performed a separate analysis to search for TF motifs in promoter regions annotated by the Mouse ENCODE project [46, 47]. OLIG2-binding motifs within ENCODE promoters were found in the upstream regulatory regions of 125 (49%) out of 254 lncRNAs that are up-regulated during the formation OPCs from NSCs. ASCL1-binding motifs inside ENCODE promoters were found in the upstream regulatory regions of 139 (63%) out of 254 lncRNAs (S6 Table). A correlation analysis was carried out to identify any correlations between the expression of TFs with binding motifs within ENCODE promoter regions upstream of the lncRNAs and the target lncRNAs. A heatmap representing the correlations between the expression of lncRNAs and TFs is included in S4 Fig.

### Loss-of-function tests reveal the functions of lncRNAs essential for OPC cell fate determination

Several criteria were applied to select prospective candidate lncRNAs for testing potential roles in OPC fate determination. First, the candidates had to be up-regulated in OPCs compared to NSCs, and substantially expressed in OPCs (e.g., FPKM > 3 in OPC). Second, lncRNAs had to be specifically enriched in OPCs among the cell types studied. Third, there had to be evidence of OLIG2 binding in the proximal regions of the lncRNA genes. These criteria narrowed our focus to specific candidates for further analysis.

**Fig 5:**
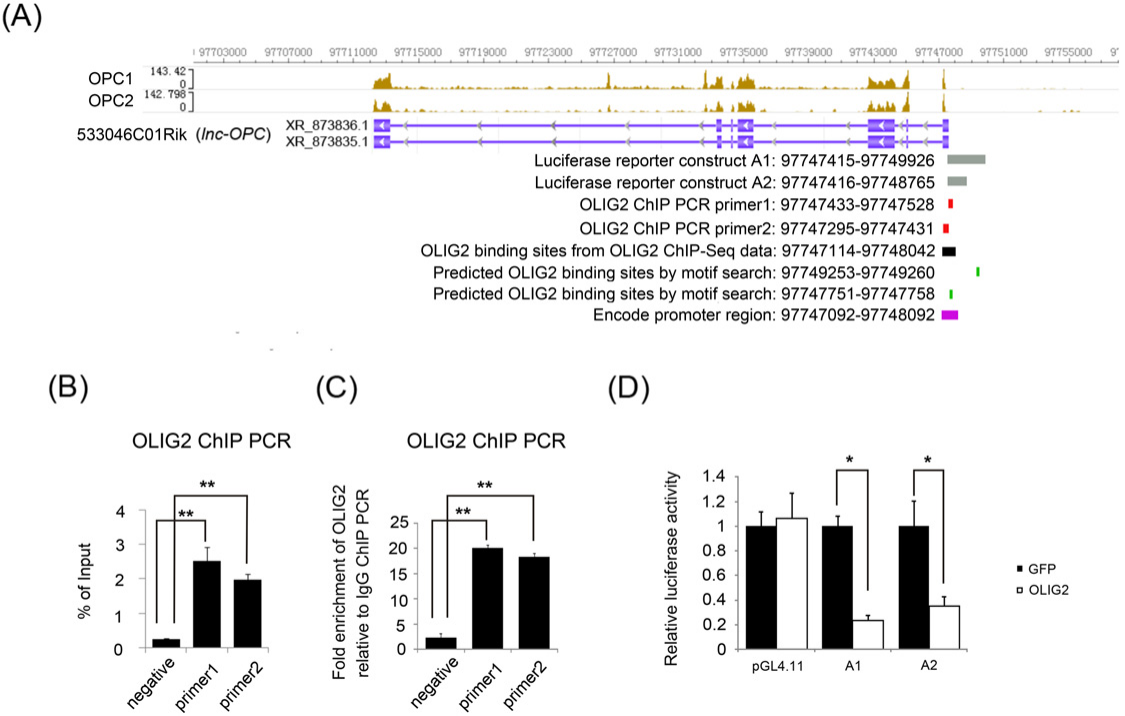
OLIG2 binds to upstream regulatory region of *lnc-OPC* and controls its expression. (A) Read mapping signal tracks of *lnc-OPC*. The upstream regulatory regions of *lnc-OPC* cloned into luciferase reporter constructs are indicated by grey bars. OLIG2 ChIP-qPCR targeted regions are indicated by red bars. OLIG2-binding sites revealed by OLIG2 ChIP-Seq are indicated by black bars. OLIG2-binding sites predicted by motif search are indicated by green bars. The ENCODE-annotated promoter region is indicated by a purple bar. (B, C) Detection of OLIG2-binding sites in the upstream regulatory region of *lnc-OPC* by OLIG2 ChIP-qPCR. Two pairs of primers were used. Enrichment over genomic input DNA and fold changes over IgG control were calculated. Experiments were performed in triplicate and error bars indicate Standard Error. *t*-test analysis ** *p*< 0.01. (D) The effect of OLIG2 on luciferase expression is represented as changes in relative luciferase activity. The 293FT cells were cotransfected with the luciferase reporter plasmids containing different lengths of the *lnc-OPC* regulatory region, along with an OLIG2- or GFP-expressing construct. Empty pGL4.11 vector was used as a control. The luciferase activity of cells cotransfected with GFP and luciferase reporter plasmids were used as controls and set to 1. Luciferase activity for each sample was normalized to GFP-transfected controls. Experiments were performed in triplicate and error bars indicate Standard Error. *t*-test analysis * *p*< 0.05.

Subsequently, we performed qPCR validation and loss-of-function shRNA knockdown experiments for these candidates to investigate their possible roles in OPC fate determination. Finally, we focused on *lnc-OPC*, because it was among the most enriched lncRNAs in OPCs, according to RNA-Seq data. Interestingly, two predicted non-coding transcripts (XR_873836.1 and XR_873835.1, part of RIKEN cDNA *5330416C01Rik* gene) were documented independently in the NCBI Reference Sequence after we had identified *lnc-OPC de novo*, which supported our analysis result (Fig 5A). As predicted by our motif search, there are OLIG2 binding sites in the upstream regulatory region of *lnc-OPC* and one is located within an ENCODE promoter. In order to test the *in silico* prediction, we performed OLIG2 ChIP-Seq and found one binding peak overlapping with these predicted motifs (Fig 5A and S7 Table). Moreover, we performed OLIG2 ChIP-qPCR using NSC cell lysates. Significant enrichment over genomic input DNA and IgG control were detected by both pairs of primers in OLIG2 ChIP samples, which indicated OLIG2 binding in the upstream regulatory region of *lnc-OPC* (Fig 5B and 5C). During CNS development, OLIG2 plays an important role in maintaining NSCs and the lineage specification of NSCs into OPCs. The observation that OLIG2 binds to the upstream regulatory region of *lnc-OPC* suggests that OLIG2 might regulate the expression of *lnc-OPC*. We created two constructs (A1 and A2) containing the upstream regulatory region of *lnc-OPC* fused to a luciferase reporter gene, as indicated in Fig 5A. To test whether OLIG2 could regulate the transcription of *lnc-OPC*, 293FT cells were cotransfected with fusion luciferase reporter constructs along with the construct expressing either OLIG2 or GFP (as a control). The expression of OLIG2 significantly inhibited luciferase expression from the fusion reporter constructs by more than 64% compared to the GFP control (by 76.55% for A1 and 64.83% for A2) (Fig 5D). However, no effect on luciferase expression was observed when cotransfecting OLIG2 and empty pGL4.11 vector control. These observations indicated that OLIG2 binds to the upstream regulatory region of *lnc-OPC* and represses the transcription of *lnc-OPC* in NSCs.

**Fig 6:**
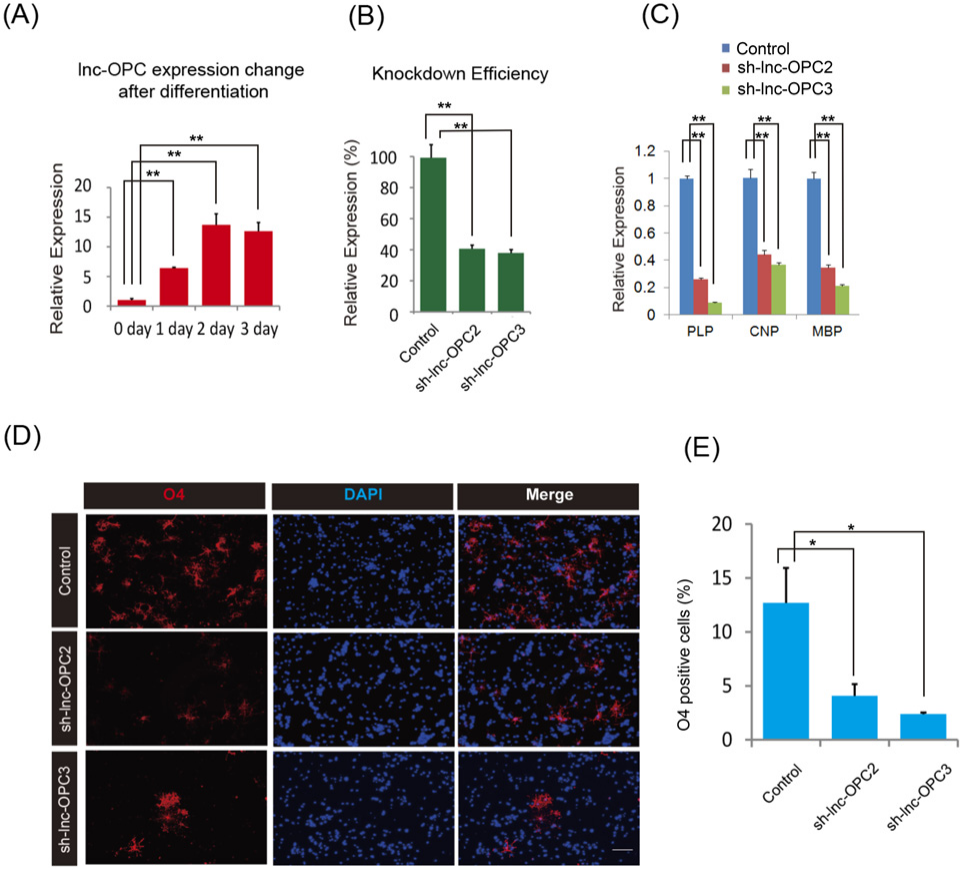
Loss-of-function experimental validation of *lnc-OPC* function in OPC formation. (A) Changes in *lnc-OPC* expression upon differentiation of OPCs from NSCs. *lnc-OPC* expression in NSCs without differentiation was set to 1. The expression of *lnc-OPC* increased upon differentiation of OPCs from NSCs. (B) Efficiency of knockdown of *lnc-OPC* expression by shRNAs in puromycin-selected NSCs, as evaluated by qPCR. Two shRNAs, sh-lnc-OPC2 and sh-lnc-OPC3, succeeded in knocking down *lnc-OPC* expression when compared to control (sh-Luc). *lnc-OPC* expression in control was set to 100%. (C) Expression of OPC markers in control (sh-Luc) and *lnc-OPC*-depleted cultures (two constructs were used: sh-lnc-OPC2 and sh-lnc-OPC3) upon differentiation of OPCs from NSCs. The expression of OPC markers in controls were set to 1. Knockdown of *lnc-OPC* reduced the expression of PLP, CNP, and MBP. For A-C, experiments were performed in triplicate and error bars indicate Standard Error. *t*-test analysis ** *p*< 0.01. (D) Immunostaining analysis of OPC formation in control (sh-Luc) and *lnc-OPC*-depleted cultures (two constructs were used: sh-lnc-OPC2 and sh-lnc-OPC3). Images were taken after differentiation for 5 days. Blue: DAPI, Red: O4. Scale bar, 100 μm. (E) Quantification of OPC differentiation from puromycin-selected NSCs was measured as the percentage of O4+ cells. Results are from three independent experiments and 10 randomly selected microscopy fields were counted each time. *t*-test analysis * *p*< 0.05.

qPCR was used to independently monitor changes in the expression of *lnc-OPC* during OPC differentiation from NSCs (Fig 6A). The expression of *lnc-OPC* was elevated gradually and greater than ten fold increase was observed 2 days after NSC differentiation. We designed three lentivirus-based short hairpin RNAs (shRNAs) to target the last exon of *lnc-OPC* and two of them succeeded in knocking down more than 50% of *lnc-OPC* expression in NSC culture (Fig 6B). Subsequently, puromycin-selected NSCs were differentiated to OPCs in culture. Knockdown of *lnc-OPC* resulted in a significant decrease in the expression of OPC markers (MBP, PLP1, CNP) and O4+ (oligodendrocyte surface marker) in cells, as assessed by qPCR and immunostaining experiments (Fig 6C, 6D and 6E). qPCR results indicated that depletion of *lnc-OPC* inhibited MBP, PLP1, and CNP expression by >60% when compared to control cells (Fig 6C). Additionally, O4-positive OPCs were significantly reduced in *lnc-OPC*-depleted cells compared to the control cells (Fig 6D and 6E). To rule out the possibility that the reduction in OPC cell number was due merely to an impaired ability of NSCs to proliferate, we performed a BrdU cell proliferation assay and found that there was no significant influence on NSC proliferation after shRNA knockdown (S5 Fig). To further confirm and examine the effect of *lnc-OPC* depletion at a genome-wide scale, we performed RNA-Seq to evaluate the transcriptome changes caused by *lnc-OPC* knockdown during OPC differentiation from NSC (S6 Fig). DAVID GO functional term enrichment analysis, using genes that are differentially expressed in the *lnc-OPC* knockdown compared to the control, revealed significant enrichment of ‘oligodendrocyte development’, ‘oligodendrocyte differentiation’, ‘glia cell development’, and ‘axon ensheathment’ terms that are associated with oligodendrogenesis (S8 Table).

Collectively, our experimental and computational analysis suggests an essential role of *lnc-OPC* in oligodendrogenesis.

## Discussion

The central nervous system is comprised of an intensively diverse array of cell types, which complicates the task of identifying cell-type specific transcripts and limits the utility of data obtained from tissue samples. A large number of lncRNAs are reported as expressed in the mouse brain [8, 10]; however, the lncRNA catalogs created using tissue or organ samples have not been comprehensive [10]. Thus, gene expression dynamics occurring in rare cell types may go undetected during analysis of these kinds of samples. Hence, direct reconstruction and measurement of the transcriptomes of specific cell types is crucial to understanding the gene dynamics that underlie cellular phenotypes. This is particularly true for measuring the expression profiles of lncRNAs that are considerably more cell-type specific than are protein-coding genes [2, 3, 15]. Our RNA-Seq data for highly purified cell types from mouse brain represent the first lncRNA expression database for collective populations of glia and neurons. Because we have been able to identify many multi-exonic lncRNAs that were not annotated previously, our study highlights the importance of using cell-type specific data for lncRNA identification and functional characterization.

The field of lncRNA functional studies is still in its early stage. Because lncRNAs are less evolutionarily conserved than protein-coding genes, analysis of their sequence conservation alone lacks the power to assess the biological significance of lncRNAs [48, 49]. To date, the functional roles of most of the characterized lncRNAs were first inferred by transcriptional profiling and co-expression analyses of lncRNAs and protein-coding genes. We havepredicted potential functions of lncRNAs using our brain cell-type specific RNA-Seq data and non-brain RNA-Seq datasets in order to prioritize candidates for functional tests. The predicted functions of those documented lncRNAs from the CNS matched well with, but were not restricted to, their functions previously described in the literature, which suggests that lncRNAs may be involved in different biological functions in different cell types. Nevertheless, association alone cannot distinguish whether the change in expression is the cause or the consequence of differences in cellular state [13]. For example, aside from known functions in the literature, the functional terms ‘Neural tube development’ and ‘Brain development’ were also predicted to be significantly associated with *Malat1*; however, recent studies have shown that *Malat1* is dispensable for CNS development [50, 51], indicating that functional predictions arising from by ‘guilt-by-association’ must be received with caution, and experimental validation is necessary. To select potential candidates for functional validation, other genomic information such as specific TFs binding is useful.

We analyzed the DNase I digital footprint datasets related to CNS development from the ENCODE project to test whether lncRNAs are under active regulation by transcription factors during CNS development. Using the size of *cis*-regulatory modules that are bound by TFs within the promoter regions as an indicator, we showed that a portion of lncRNAs have larger *cis*-regulatory modules than do random intergenic regions or even some TFs. Furthermore, a large number of lncRNAs were associated with △DHSs across the four ENCODE datasets studied. Neighboring △DHSs activated in different developmental contexts are enriched for specific TF sequence motifs that are known to be associated with the respective cellular functions. Taken together, the results of these analyses indicate that lncRNAs are dynamically controlled by TFs that specify CNS development.

Investigating the molecular mechanisms underlying OPC formation is critical in understanding oligodendrocyte cell fate determination and for harnessing OPCs for cell-based therapies in regenerative medicine. Although many protein-coding and microRNA genes have been shown to play a critical role in oligodendrogenesis, functional characterization of lncRNAs during OPC fate determination has not been carried out systematically. A previous study employing a custom-designed microarray also showed that a number of known lncRNAs exhibit dynamic expression patterns during oligodendrocyte lineage specification, suggesting that they may play a role in neural stem cell fate decisions and oligodendrocyte lineage maturation [6]. However, incomplete annotation of lncRNAs hindered interrogation of novel lncRNAs and thus conclusions regarding functional significance were limited. Our study identified hundreds of lncRNAs that are regulated during NSC-to-OPC differentiation, and a substantial fraction of them are under the control of key TFs associated with oligodendrogenesis. Combining the TF binding site information, differential expression analysis, and enrichment analysis, lncRNAs that are highly enriched in OPC were identified as potential candidates involved in OPC fate determination. Depletion of the most enriched lncRNA in OPC, *lnc-OPC*, resulted in reduced OPC formation, suggesting that *lnc-OPC* plays an essential role in the circuitry that controls oligodendrogenesis. Intriguingly, ChIP-Seq and luciferase assay results suggested that OLIG2 binds to the upstream regulatory region of *lnc-OPC* and represses the transcription of *lnc-OPC* in NSC. Further studies will be necessary to elucidate the molecular mechanism through which the repression of *lnc-OPC* transcription is released during the formation of OPC from NSC and how *lnc-OPC* acts to modulate oligodendrogenesis. Previous studies have indicated that lncRNAs can serve as modular scaffolds for chromatin modifying complexes that modulate the epigenetic landscape during cell fate determination [14, 52]. Such a mechanism could operate in *lnc-OPC* function. Although the focus of our study is on the lncRNAs that are up-regulated in OPC compared to NSC, the possibility of functional roles for down-regulated lncRNAs is not ruled out. Some of these lncRNAs may function as inhibitors of OPC formation and may help maintain the self-renewal ability of NSCs [12].

**Fig 7:**
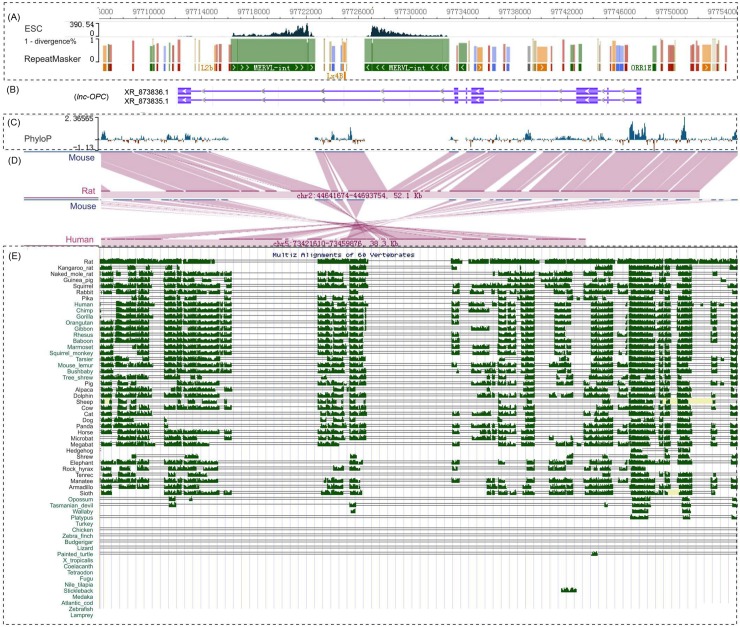
Comparative genomics analysis of *lnc-OPC* revealed its evolutionary history. (A) Signal tracks of the expression of two TEs at the *lnc-OPC* locus in ESCs. RepeatMask annotation (bottom) shows two MERVL located in the last intron of *lnc-OPC*. The height of the bar above the repeat elements corresponds to (1 –%divergence). (B) The structure of *lnc-OPC* is shown. (C) Conservation displayed as Phylip score. (D) Pairwise alignments of mouse-rat and mouse-human *lnc*-OPC. (E) Multiple alignments of conservation of *lnc-OPC* in 60 vertebrates shown in the UCSC browser.

Inspecting the multiple alignments of *lnc*-OPC for 60 vertebrate species in the UCSC genome browser revealed remarkable sequence conservation of the *lnc-OPC* loci in placental mammals. These multiple alignments, as well as pairwise alignments, indicate two inserted segments located in the last intron of *lnc-OPC* in the mouse (Fig 7). These two insertions were not found in rat and other placental mammals, suggesting they are mouse-lineage specific and were introduced after the divergence of the lineages leading to mouse and rat. Interestingly, the inserted segments were highly expressed in ESCs. The symmetrical expression patterns of these two segments indicate that they may be repetitive elements. Indeed, we found that two MERVL (mouse endogenous retroviral element) transposable elements (TEs) perfectly matched the RepeatMasker annotation retrieved from the UCSC genome browser.

About two out of three lncRNA transcripts cataloged in zebrafish, mouse, and human are estimated to contain at least one TE-derived sequence, whereas these sequences are rarely found in protein-coding genes [53]. TEs have been shown to contribute signals essential for the biogenesis of many lncRNAs by influencing their transcription initiation, splicing, or polyadenylation [53, 54]. In particular, TEs embedded in introns can influence transcription of the host gene, causing upstream transcript polyadenylation, thus promoting the possibility of alternative polyadenylation or alternative splicing [55, 56]. In this case, the two TEs are located in the last intron of *lnc-OPC*. There are several annotated shorter transcripts upstream of the TEs (e.g., XR_873837.1, XR_873834.1, and XR_873838.1) that overlap with the 5’ part of *lnc-OPC*. Therefore, the TE insertions may have caused alternative splicing or alternative polyadenylation and contributed to the evolution of transcript diversity at the *5330416C01Rik* gene loci. Additionally, TEs, particularly LTR/ERVs (LTR: long terminal repeat; ERV: endogenous retroviral), in the vicinity of lncRNA genes can be involved in the regulation of their transcription and contribute to tissue-specific expression profiles [53, 57, 58]. The two MERVL TEs have higher expression signals in various other cell types than in OPC. Thus, it is possible that these TEs may be involved in *lnc-OPC* evolution and transcriptional control. This hypothesis and the detailed mechanisms require further investigation.

Multiple sclerosis is a chronic inflammatory demyelinating disease that involves the loss of oligodendrocytes. Subventricular zone (SVZ)-derived progenitors can be activated and repopulate the neural lesions after demyelination insults in multiple sclerosis. We analyzed the data from a previous study using experimental allergic encephalomyelitis (EAE) mouse models (GSE47486) [59]. Interestingly, *5330416C01Rik* gene (*lnc-OPC*) was found to be up-regulated more than seven fold in glial progenitor cells (NG2^+^ cells) from the SVZ of the EAE models compared to those from wild-type mice, suggesting that *lnc-OPC* may be involved in this disease (S7 Fig).

It should be noted that the lncRNA annotation used in our study was retrieved from multiple sources including GENCODE, RefSeq, Ensembl, lncRNAdb, and collections of lncRNAs identified by several other groups. We identified lncRNAs *de novo* from purified brain cell types and the coding potential of identified non-coding RNAs was evaluated using the Coding-Potential Assessment Tool (CPAT). However, a recent study identified products of non-canonical translation from RNAs from mouse neurons that had been classified as non-coding [60]. Thus such a possibility should be considered. Another aspect that we should point out is that only polyadenylated RNAs were selected for RNA-seq library construction in the present study. However, polyA-selected RNAs account for only a portion of non-ribosomal, non-mtRNA [61]. Future investigation using non-polyadenylated RNAs for RNA-seq library construction could help to identify and reveal the functions of non-coding RNAs that are not polyadenylated.

Altogether, our study produced a rich and unprecedented database of lncRNA expression by various purified cell types from mouse brain. The integrative analysis framework that we established in the present study can serve as a model for investigating functional lncRNAs in other cell types.

## Materials and Methods

### Neural stem cell culture and differentiation

Mouse neural stem cells from the cortex of embryonic (E14.5) CD-1 mice were purchased from R&D Systems. Briefly, cells were cultured and passaged every 2 days as monolayers in MEM/F12 medium containing Glutamax, non-essential amino acids, B27, N2 supplement, 20 ng/ml EGF, and 20 ng/ml FGF. Cells were dissociated using Accutase (Invitrogen) and seeded onto poly-d-lysine-coated plates or dishes. NSCs were differentiated into OPCs in DMEM/F12 medium containing Glutamax, non-essential amino acids, BSA, B27, N2 supplement, 20 ng/ml CNTF, and 40 ng/ml T3. For immunostaining, NSCs were allowed to differentiate for 5 days. For mRNA extraction and qPCR, NSCs were allowed to differentiate for 3 days.

### RNA extraction, cDNA synthesis, and Quantitative Real-Time PCR (qRT-PCR)

RNA extraction was performed using TRIzol reagent, then DNase-treated RNA was reverse transcribed with the iScript cDNA Synthesis Kit according to the instructions from the manufacturer (Bio-Rad). Quantitative Real-Time PCR (qRT-PCR) was performed using reactions prepared with the SYBR Green Master Mix (Bio-Rad) performed on the ABI PRISM 7900HT Sequence Detection System. Relative gene expression was calculated by the 2^-ΔΔCT^ method using GAPDH as the reference gene.

### ChIP (chromatin immunoprecipitation)-qPCR and ChIP-Seq

ChIP was performed using 10^7^ mouse NSCs per reaction. Cells were dissociated by treatment with Accutase and cross-linked in 1% (vol/vol) formaldehyde for 10 min at RT with rotation. Then, 0.125 M glycine was used to quench the cross-linking reaction. Cells were pelleted and washed with ice-cold PBS. Next, nuclei were isolated and a Bioruptor sonicator (Diagenode) was used to shear chromatin DNA. Either 5 μl of OLIG2 antibody (AB9610, Chemicon) or 5 μl of normal rabbit IgG (#2729, Cell Signaling) was added to Dynabeads Protein A (Invitrogen) beads and incubated for 3 h at 4°C with rotation. Then, the Dynabeads-antibody complexes were incubated with sheared chromatin DNA overnight at 4°C. After immunoprecipitation, the precipitated complex was treated with RNase A and Proteinase K, and incubated at 65°C overnight to reverse crosslinks. Primers were designed and qRT-PCR experiments were performed. ChIP-qPCR data was normalized either by the Percent Input method or relative to the IgG control. The ChIP-Seq library was constructed by using DNA SMART ChIP-Seq Kit according to the manufacturer's instructions (Clontech) and was sequenced on the Illumina HiSeq 2000 Sequencer. Input chromatin sample was prepared in parallel with OLIG2 ChIP sample. The generated reads were mapped to the mouse genome mm10 using Bowtie version 0.12.7 and MACS version 1.4.2 was used to call peaks for ChIP-Seq data [62]. The output from MACS was filtered using the following more stringent criteria: (1) *p* value cutoff <10^−9^; (2) fold enrichment > five fold (3) tag number >20.

### RNA-seq library construction and sequencing

RNA-seq was performed using the same procedures as described previously [15, 20].

### shRNA knockdown of lncRNAs

At least three shRNAs against *lnc-OPC* were separately cloned into a pLKO.1 lentiviral vector and recombinant lentivirus was produced in 293FT cells. NSCs were transduced with recombinant lentivirus after 24 h of culture on poly-d-lysine pre-coated plates. Non-infected cells were eliminated using fresh culture medium containing 0.5 μg/ml of puromycin 1 day after infection. Infected cells were proliferated for 3–4 days and subsequently used for the assessment of knockdown efficiency and oligodendrocyte lineage commitment.

### Immunostaining

For the oligodendrocyte surface membrane antigen O4, cells were cultured in 24-well plates and were stained with the primary antibody O4 produced by hybridoma (from Dr. Qilin Cao, personal communication) at RT for 1 h. Cells were then fixed in 4% paraformaldehyde (vol/vol) and subsequently stained using Alexa Fluor 594 goat anti-mouse IgG (H+L) antibody. DAPI was used to stain the nuclei. Three independent experiments were carried out and cell number counts were obtained from 10 randomly selected fields in each experiment.

### BrdU cell proliferation assay

Puromycin-selected NSC cells were incubated in medium with BrdU labeling reagent (Invitrogen) for 60 min and subsequently fixed in 70% ethanol for 20 min at RT. After three washes with PBS, cells were treated with 1.5 M HCl for 30 min. Cells were then immunostained with BrdU mouse antibody (Cell Signaling), Alexa Fluor 488 goat anti-mouse IgG (H+L) antibody (Invitrogen), and DAPI. Three independent experiments were carried out and the results from 10 random fields for each experiment were obtained.

### Luciferase assay

The *lnc-OPC* -luciferase reporter constructs were created by cloning 1.3- to 2.5-kb fragments upstream of the *lnc-OPC* transcription start site into the *Kpn*I and *Xho*I multiple cloning sites of the pGL4.11 vector. The constructs were cotransfected with pmaxGFP or the mouse OLIG2 expression plasmid pCMV-SPORT6.1-OLIG2 using Lipofectamine 3000 (Invitrogen) in 293FT cells. Cells were harvested and examined for luciferase activity 2 days after transfection using a Luciferase Assay System kit (Promega). Luciferase activity was measured using a Tecan Microplate Reader infinite M1000 and was normalized by the protein concentrations of each sample.

### RNA-Seq data analysis

RNA-Seq analysis was performed as described previously [15]. The Tuxedo suite was used for read mapping and transcript assembly [22].

### The prediction of lncRNA functions

RNA-Seq data from eight brain cell types and seven non-brain tissues were used for analysis (pericytes were excluded because of their relatively lower purity). RNA-Seq data from seven non-brain mouse tissues (thymus, testis, kidney, liver, lung, spleen, and heart) were obtained from the Mouse ENCODE project. Data were downloaded from GeneExpression Omnibus (GEO accession number GSE36025) and processed using the same pipeline as for brain cells. A previously described ‘guilt-by-association’ method was adopted for function prediction [4].

### Co-expression gene module construction

Weighted Gene Co-expression Network Analysis (WGCNA) R package was used to identify gene co-expression modules [63].

### Gene Set Enrichment Analysis (GSEA)

GSEA analysis was performed using the GSEA command line executable file downloaded from http://www.broadinstitute.org/gsea/index.jsp [26].

### The identification of TF binding from DNase-DGF data and ENCODE promoters

Potential TF-binding sites were determined using Find Individual Motif Occurrences (FIMO) version 4.6.1, setting the p-value threshold to 10^−5^ and using defaults for other parameters [64]. The Bayesian method, CENTIPEDE, was adopted to infer the binding status of TF binding sites in the DNase-DGF data [34]. In addition to DNase-DGF, we performed motif searches in the promoter regions annotated by the Mouse ENCODE project upstream of the lncRNAs of interest.

### The identification of dynamic DHSs

We defined dynamic DHSs as the DHSs that are enriched > three fold under one condition compared to average signals across all conditions, with signal > 10 under the enriched condition.

### *De novo* TF motif identification

For TFs that have public ChIP-Seq data, but that are not present in motif databases, we conducted *de novo* motif discovery using the MEME-ChIP included in the MEME suite [65].

### The enrichment of TF motifs in dynamic DHSs

We used the Homer software suite to test whether TF motifs are enriched in dynamic DHSs that are activated under different conditions. A binominal distribution was used to calculate *p*-values for the significance of enrichment [66].

### Differential Expression Gene (DEG) analysis

DEGs were called using the DESeq R package [67]. Only genes with > two fold change in expression and FDR < 0.05 were considered as DEGs.

### Comparative genome analysis

The UCSC genome browser was used for inspecting multiple alignments and RMSK annotations. The Epigenetics browser was used to depict the track plots [68].

## Supporting Information

S1 Fig Choosing the soft-thresholding power for WGCNA analysis. The choice of soft thresholding power was based on the criterion of approximate scale-free topology [69]. We chose the power 10, which is the lowest power for which the scale-free topology fit index curve flattens out upon reaching a high value. The left panel shows the scale-free fit index (y-axis) as a function of the soft-thresholding power (x-axis).The right panel displays the mean connectivity (degree, y-axis) as a function of the soft-thresholding power (x-axis).
(TIF)

S2 Fig Visualizing gene co-expression network as a heatmap. The heatmap depicts the Topological Overlap Matrix (TOM) among all genes used in the analysis [69]. Light color indicates low overlap and darker red color represents higher overlap. Blocks of darker colors along the diagonal are the modules. The dendrogram and module assignment (labeled in different colors in the color bar) are shown along the left and top sides.
(TIF)

S3 Fig A selected set of transcription factor footprints detected by DNase-DGF in ESCs and in Whole Brain E14.5 sample. Displayed is the aggregate signal of the DNase-DGF cutting profiles for the indicated transcription factors. The profiles were computed using CENTIPEDE on the genome-wide sets of sites that match the corresponding motif. DNase cutting sites within +/-100 bp of the motif boundary were calculated. The vertical dashed lines indicate the boundaries of the motifs.
(TIF)

S4 Fig lncRNA-TF expression correlation heatmap. Correlation analysis was carried out to identify any correlations between the expression of TFs with binding motifs inside the ENCODE promoter regions upstream of lncRNAs that are up-regulated during OPC formation and their target lncRNAs. The TFs with more binding motifs are listed toward the left side of the map. Red represents positive expression correlation and blue represents negative expression correlation. White represents no correlation.
(TIF)

S5 Fig Assay of cell proliferation by BrdU staining. The percentage of BrdU positive cells under each condition was calculated. No significant difference was observed between control and shRNA knockdowns.
(TIF)

S6 Fig Assessing global effect of *lnc-OPC* knockdown using differential expression analysis. The R package DESeq was adopted to call differentially expressed genes. The left panel is the MA-plot showing normalized mean compared to log2-fold change for the *control* compared to *lnc-OPC-depleted* sample. Red dots represent genes called as differentially expressed genes. The right panel is a histogram of *p*-values calculated by negative binomial test.
(TIF)

S7 Fig The values for the expression of *5330416C01Rik* in healthy and EAE mice were extracted from a previous study [59]. Fold changes in *5330416C01Rik* expression in EAE mice compared to healthy mice are shown in glial progenitors and neuronal progenitors.
(TIF)

S1 Table Cell purity estimation. Signals from other cell types were estimated using established cell-type specific markers.
(XLSX)

S2 Table Master table of lncRNA expression. lncRNA expression level (FPKM) calculated using Cufflinks (version 1.3.0) [22]. All analyses were performed before the *5330416C01Rik* cDNA annotation update when the *5330416C01Rik* gene did not include *lnc-OPC*.
(XLSX)

S3 Table Association matrix of lncRNAs and functional gene sets. Functional gene sets (columns) and lncRNAs (rows) are shown as positively (>0), negatively (<0), or not associated (0). NES value (normalized enrichment score) were calculated using GSEA. Only associations with FDR < 0.25 are presented in the matrix [26].
(XLSX)

S4 Table Gene Ontology (GO) enrichment analysis of co-expression modules identified by WGCNA. Each worksheet contains the analysis result calculated using the DAVID bioinformatics tool [70]. The worksheets are ordered by module size.
(XLSX)

S5 Table WGCNA clusters of protein-coding genes and lncRNAs. Module assignment for each gene is presented in the ‘Label.Merged’ column. The membership values of each gene associated with each module (color coded by WGCNA) are presented in the table.
(XLSX)

S6 Table Specific TF binding sites in proximity to up-regulated lncRNAs (OPC compared with NSC). TF occupancy was examined for each lncRNA that is up-regulated in OPC compared to NSC. The presence of TF binding in proximity to a lncRNA is indicated as ‘1’. Binding sites for OLIG2 and ASCL1 from DNAse-DGF data are shown. Annotated ENCODE promoter regions containing OLIG2 and ASCL1 binding motifs and OLIG2 ChIP-Seq result are also depicted.
(XLSX)

S7 Table OLIG2 binding peaks called by MACS from OLIG2 ChIP-Seq experiment. (XLSX)

S8 Table Differentially expressed genes called by DESeq after the depletion of *lnc-*OPC, and gene ontology (GO) functional term enrichment analysis. (XLSX)

S1 Text Supplementary methods. (DOCX)
