# Supplementary figures and images for: Comprehensive Identification of Long Non-coding RNAs in Purified Cell Types from the Brain Reveals Functional LncRNA in OPC Fate Determination
Source: PLoS Genet. 2015 Dec 18;11(12):e1005669. doi: 10.1371/journal.pgen.1005669 (PMC4980008; doi:10.1371/journal.pgen.1005669)

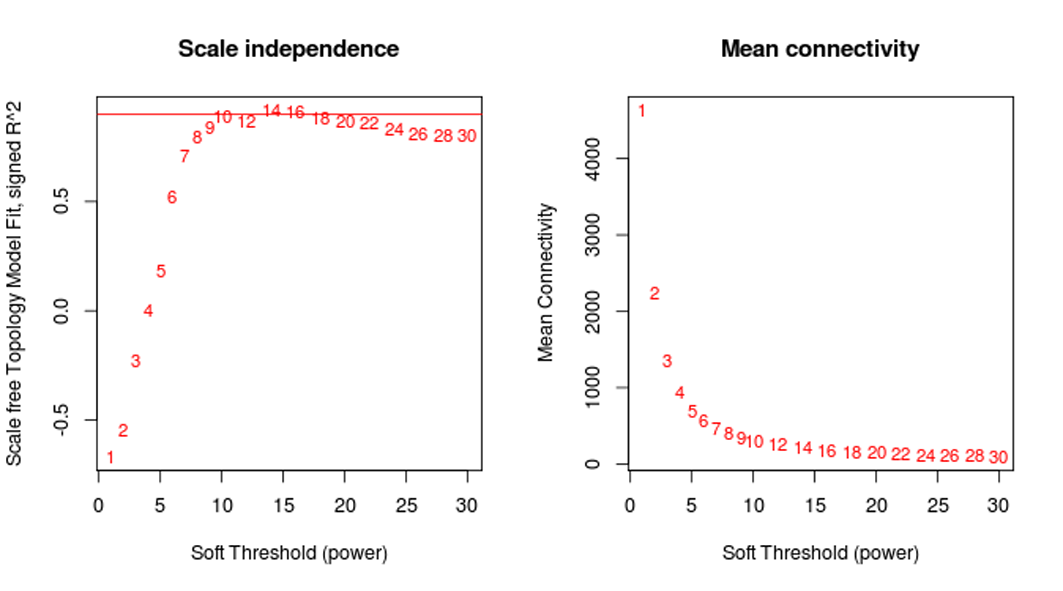

Supplement: S1 Fig — The choice of soft thresholding power was based on the criterion of approximate scale-free topology [69]. We chose the power 10, which is the lowest power for which the scale-free topology fit index curve flattens out upon reaching a high value. The left panel shows the scale-free fit index (y-axis) as a function of the soft-thresholding power (x-axis).The right panel displays the mean connectivity (degree, y-axis) as a function of the soft-thresholding power (x-axis). (TIF) [file pgen.1005669.s001.tif]

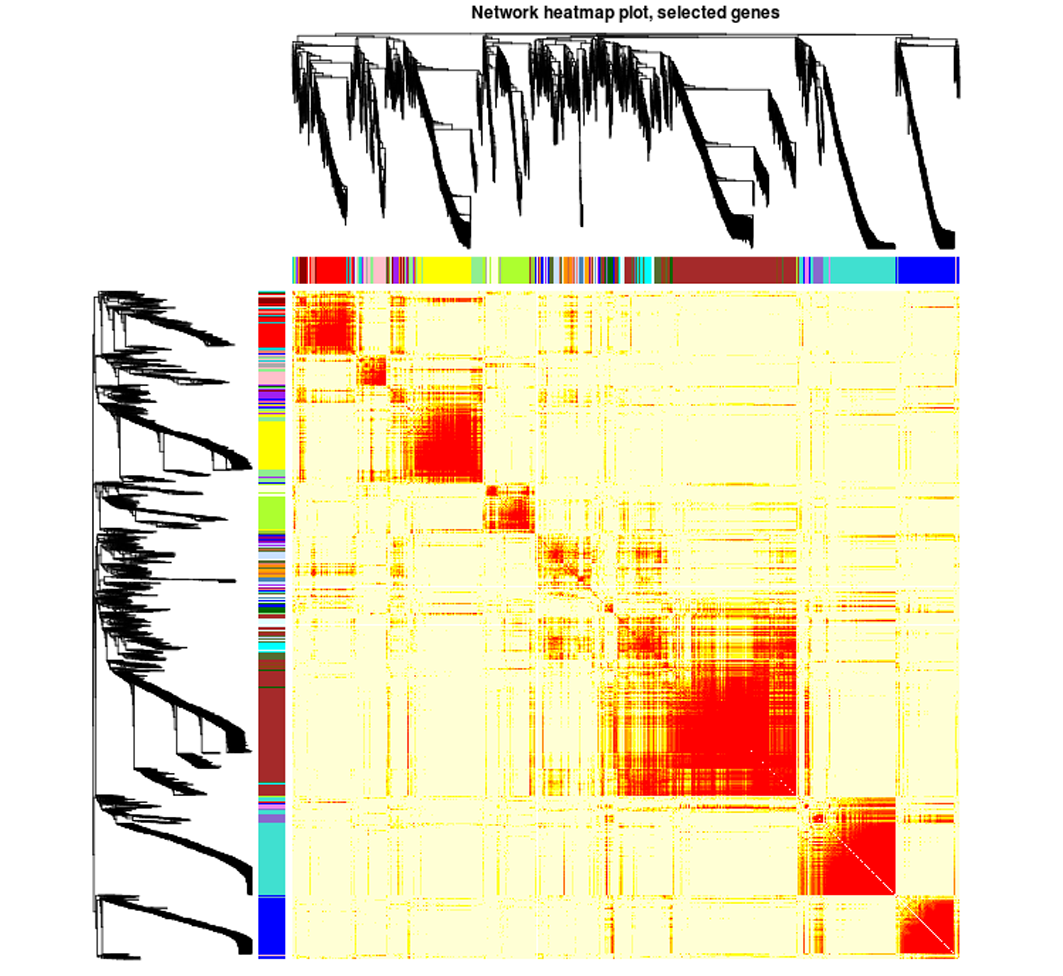

Supplement: S2 Fig — The heatmap depicts the Topological Overlap Matrix (TOM) among all genes used in the analysis [69]. Light color indicates low overlap and darker red color represents higher overlap. Blocks of darker colors along the diagonal are the modules. The dendrogram and module assignment (labeled in different colors in the color bar) are shown along the left and top sides. (TIF) [file pgen.1005669.s002.tif]

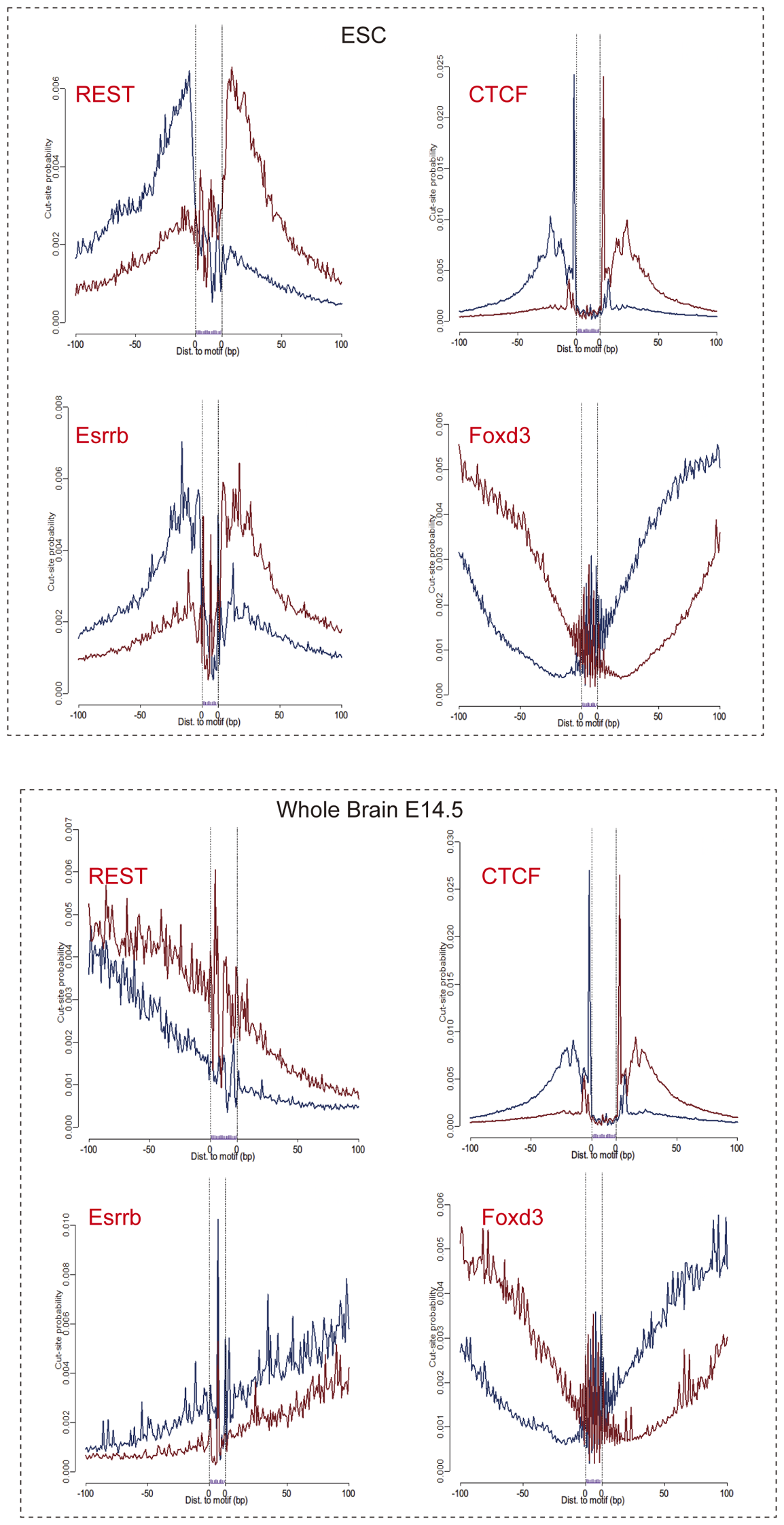

Supplement: S3 Fig — Displayed is the aggregate signal of the DNase-DGF cutting profiles for the indicated transcription factors. The profiles were computed using CENTIPEDE on the genome-wide sets of sites that match the corresponding motif. DNase cutting sites within +/-100 bp of the motif boundary were calculated. The vertical dashed lines indicate the boundaries of the motifs. (TIF) [file pgen.1005669.s003.tif]

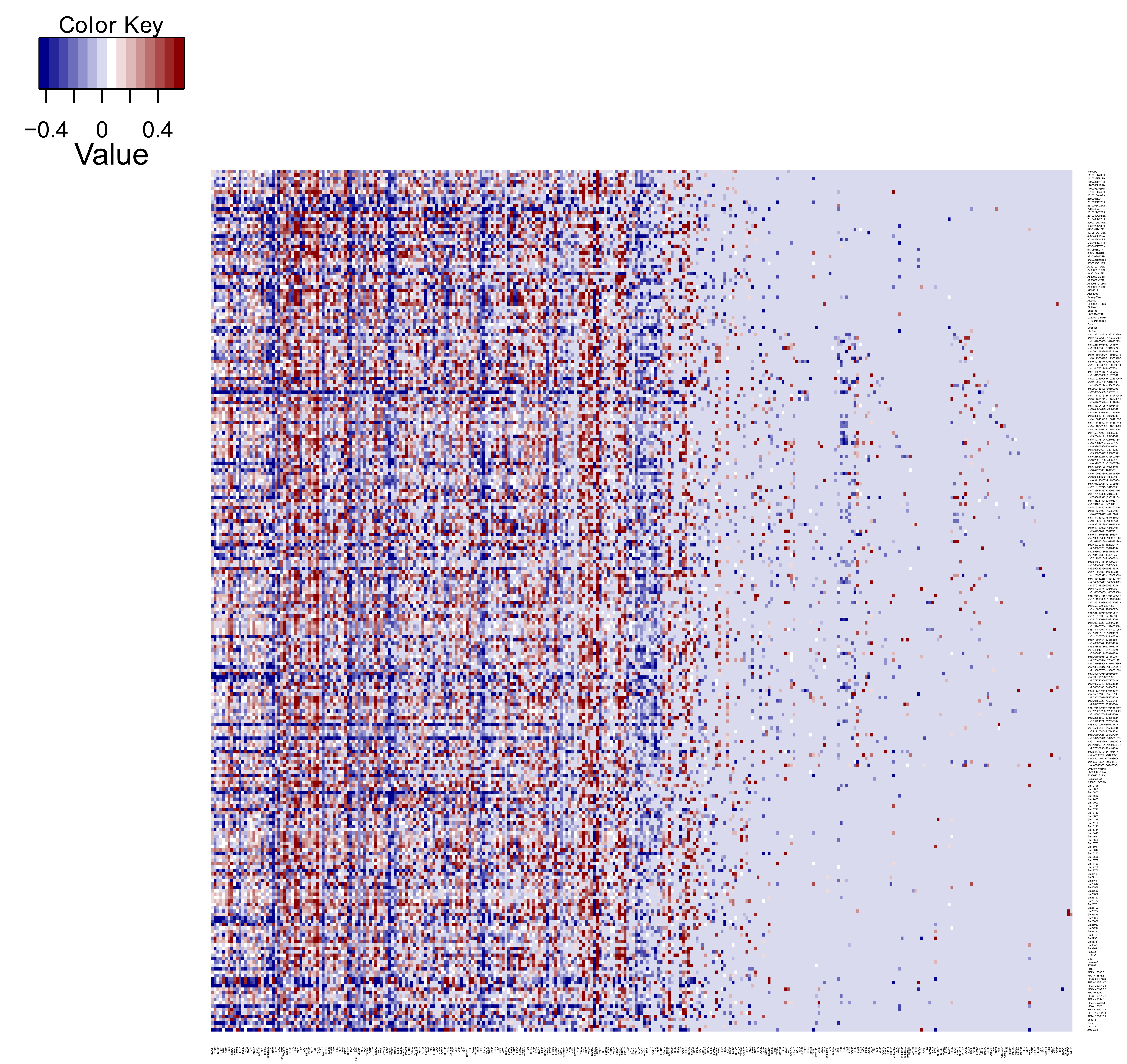

Supplement: S4 Fig — Correlation analysis was carried out to identify any correlations between the expression of TFs with binding motifs inside the ENCODE promoter regions upstream of lncRNAs that are up-regulated during OPC formation and their target lncRNAs. The TFs with more binding motifs are listed toward the left side of the map. Red represents positive expression correlation and blue represents negative expression correlation. White represents no correlation. (TIF) [file pgen.1005669.s004.tif]

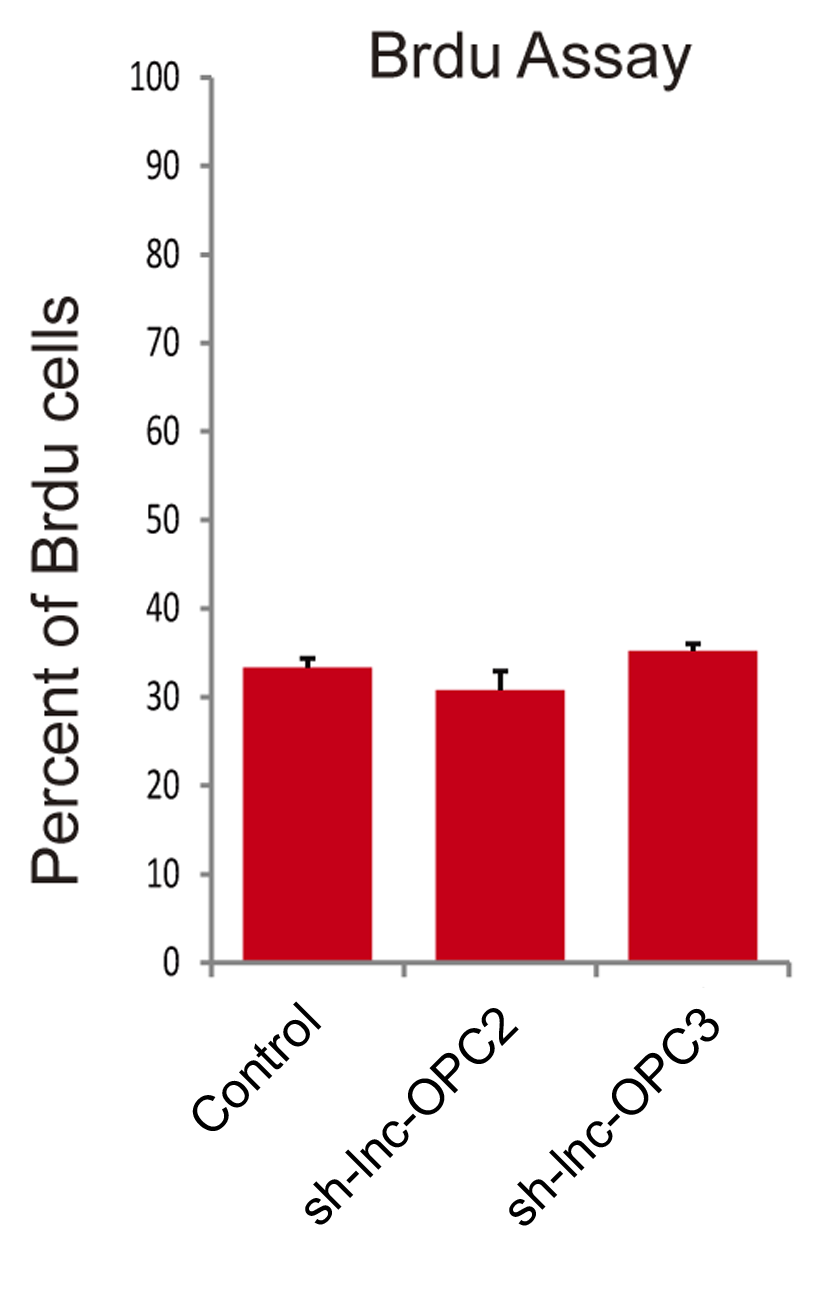

Supplement: S5 Fig — The percentage of BrdU positive cells under each condition was calculated. No significant difference was observed between control and shRNA knockdowns. (TIF) [file pgen.1005669.s005.tif]

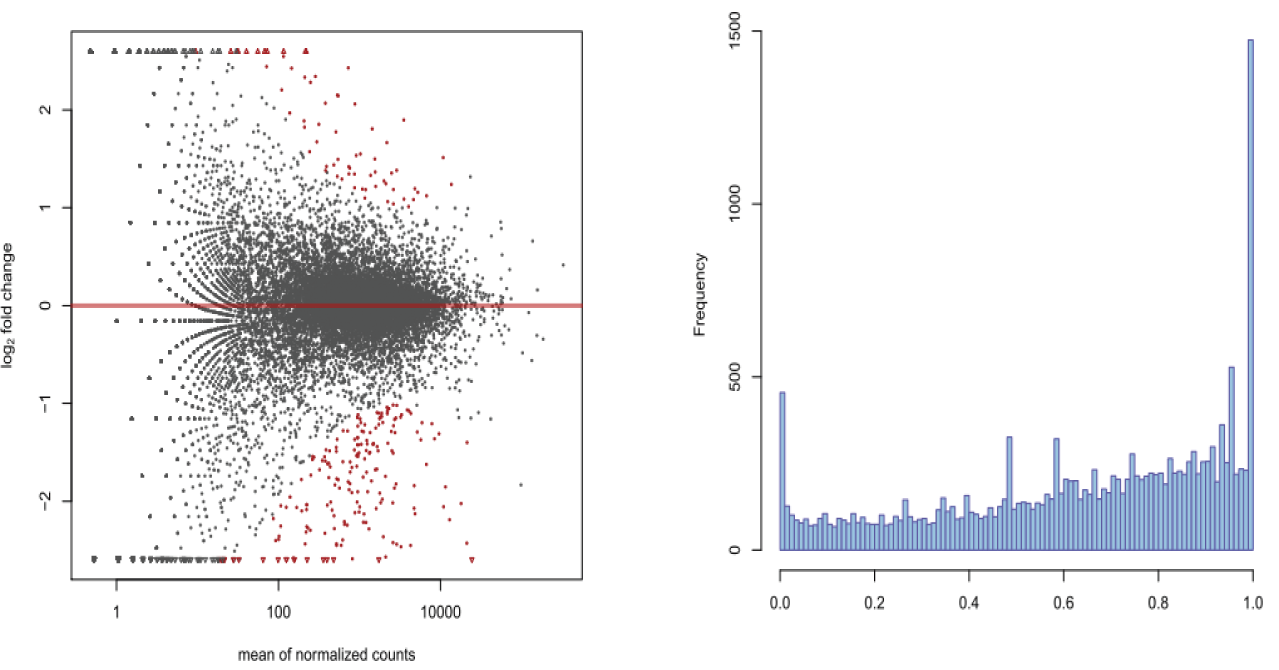

Supplement: S6 Fig — The R package DESeq was adopted to call differentially expressed genes. The left panel is the MA-plot showing normalized mean compared to log2-fold change for the control compared to lnc-OPC-depleted sample. Red dots represent genes called as differentially expressed genes. The right panel is a histogram of p-values calculated by negative binomial test. (TIF) [file pgen.1005669.s006.tif]

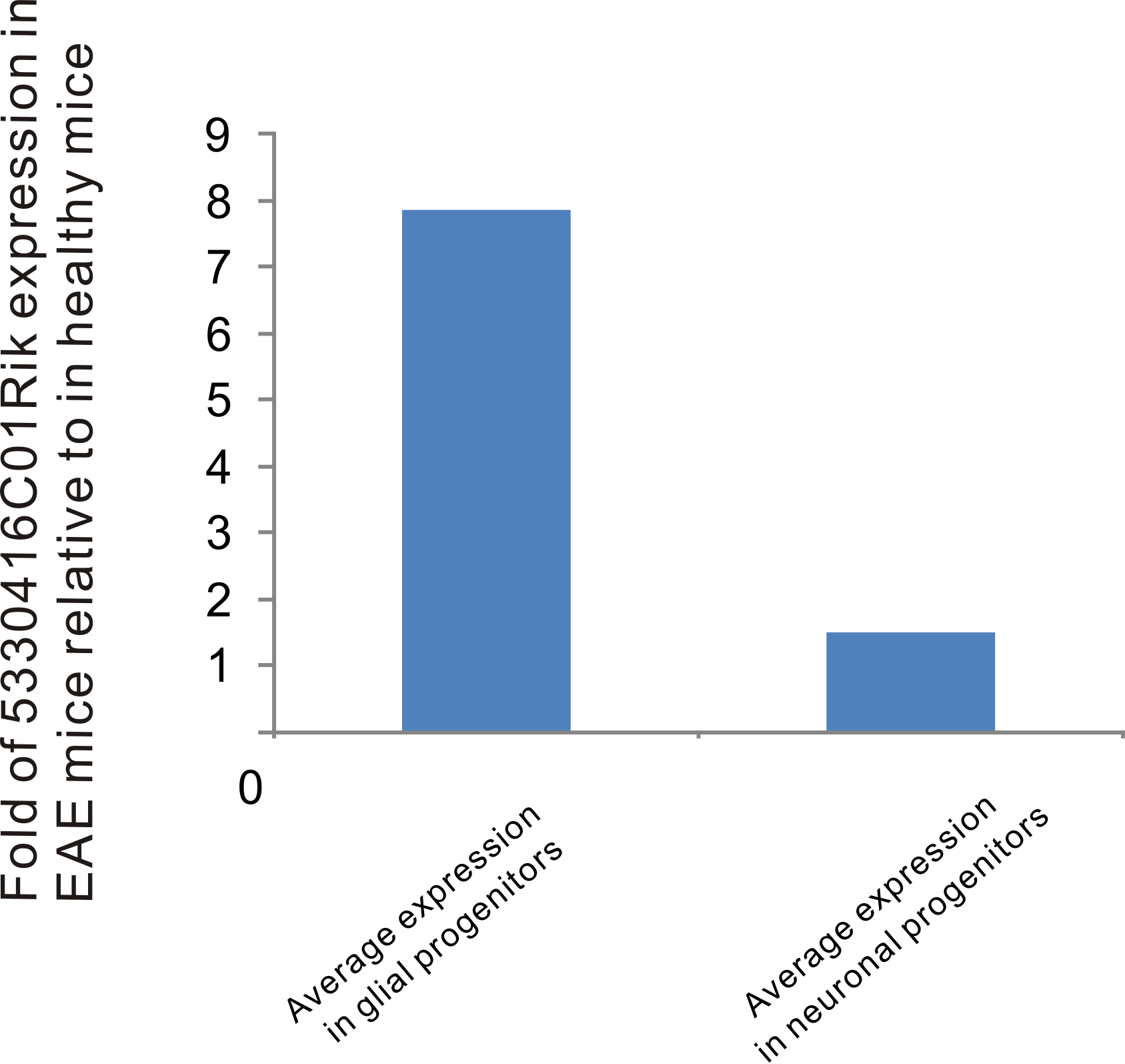

Supplement: S7 Fig — Fold changes in 5330416C01Rik expression in EAE mice compared to healthy mice are shown in glial progenitors and neuronal progenitors. (TIF) [file pgen.1005669.s007.tif]
