# Supplementary material for: Comprehensive Identification of Long Non-coding RNAs in Purified Cell Types from the Brain Reveals Functional LncRNA in OPC Fate Determination
Source: PLoS Genet. 2015 Dec 18;11(12):e1005669. doi: 10.1371/journal.pgen.1005669 (PMC4980008; doi:10.1371/journal.pgen.1005669)
Supplement: S1 Text — (DOCX) [file pgen.1005669.s016.docx]

**Supplementary Information**

**Comprehensive Identification of Long Non-coding RNAs in Purified Cell Types from the Brain Reveals Functional LncRNA in OPC Fate Determination**

Xiaomin Dong^1,2^, Kenian Chen^1,2^, Raquel Cuevas-Diaz Duran^1,2^, Yanan You^1,2^, Steven A. Sloan^3^, Ye Zhang^3^, Shan Zong^1,2^, Qilin Cao^1,2^, Ben A. Barres^3^, and Jia Qian Wu^*1,2^

**Supplementary Methods**

**RNA-Seq Library Construction and Sequencing**

RNA-seq was performed using the procedures described previously [[1](#_ENREF_1), [2](#_ENREF_2)]. Primary mouse neural stem cells (mNSC) were sequenced using the same procedures as described previously and data were deposited into GEO. Briefly, the polyadenylated fraction of RNA isolated from cells was used to obtain 100-bp paired-end RNA-seq. Sequence quality was assessed using the Agilent 2100 Bioanalyzer. Samples with high RNA integrity numbers [(>8)] were used for library construction. One hundred nanograms of RNAs were used to construct each sequencing library. A TruSeq RNA Sample Prep Kit (Illumina) was used to construct poly(A)-selected paired-end sequencing libraries following the TruSeq RNA Sample Preparation V2 Guide (Illumina). All libraries were sequenced on the Illumina HiSeq 2000 Sequencer.

**RNA-Seq Data Collection**

RNA-Seq data from purified cell types from mouse cortex, including neurons (N), astrocytes (A), oligodendrocyte precursor cells (OPC), newly formed oligodendrocytes (NFO), myelinating oligodendrocytes (MO), microglia (MGL), endothelial cells (Endo), pericytes (Peri), as well as data from mouse whole cortex tissues were retrieved from our previous work and are available at the National Center for Biotechnology Information (NCBI) Gene Expression Omnibus (GEO) data repository (accession number: GSE52564) [[1](#_ENREF_1)]. RNA-Seq data for mouse embryonic stem cells (mESCs) was downloaded from GEO (accession number: GSE20851). Mouse ENCODE RNA-Seq data from other tissues (thymus, testis, kidney, liver, lung, spleen, and heart) were obtained from GEO (accession number: GSE36025).

**Transcriptome Construction and lncRNA Identification**

Read mapping, transcript assembly, and expression estimation were performed as described in our previous publications [[1](#_ENREF_1), [2](#_ENREF_2)]. We mapped the paired-end reads to the mouse reference genome (UCSC version mm10) using TopHat (version 2.0.11) and Bowtie2 (version 2.2.2) [[3](#_ENREF_3), [4](#_ENREF_4)]. Cufflinks (version 1.3.0) was used to assemble transcripts from genomic read alignments. TopHat was run using default settings, except using the option “-G mm10_genes.gtf” (mm10_genes.gtf is an annotation file within the Illumina iGenome package, downloaded from http://cufflinks.cbcb.umd.edu/igenomes.html). Cufflinks was run using default settings without any annotation file to output a .gtf that contains structure information for assembled known and novel transcripts from each sample. To identify novel transcripts, transcripts from each cell type were first assembled using Cufflinks. All transcripts were then merged using Cuffmerge and then compared to a reference annotation file (mm10_genes.gtf) using Cuffcompare [[5](#_ENREF_5)]. Transcripts not matching any reference transcripts were given a class code ‘u’ (to designate them as unknown intergenic transcripts) by Cuffcompare and the considered as ‘novel’ transcripts not present in the reference file. The resulting .gtf was then filtered and only multi-exonic transcripts were retained [[6](#_ENREF_6), [7](#_ENREF_7)]. A recently developed Coding-Potential Assessment Tool (CPAT: http://lilab.research.bcm.edu/cpat/) was used to discriminate potential non-coding transcripts from coding transcripts. Novel transcripts labeled as ‘non-coding’ by CPAT were retained as ‘novel’ (compared to reference) lncRNAs [[8](#_ENREF_8)].

In order to generate a comprehensive lncRNA annotation for downstream analysis, we surveyed available lncRNA annotations in the public domain. lncRNA annotations were retrieved from well-known canonical databases, including lncRNAdb, GENCODE, Ensembl, and UCSC RefSeq genes. For lncRNAdb, the transcript structures were obtained using the BLAT program when only sequence information was available. We also added several recently published RNA-Seq datasets related to brain or CNS development to our analyses, including RNA-Seq data from subventricular zone (SVZ) tissue and olfactory bulb (OB), among others [[7](#_ENREF_7), [9-11](#_ENREF_9)]. To combine lncRNA annotations generated using RNA-Seq, we merged lncRNA transcripts identified from the same loci that were not annotated by GENCODE, or any other canonical lncRNA database, into single transcripts because distinguishing full-length isoforms from partially reconstructed fragments is not always possible without further experimental evidence, especially when sequencing depths vary among different RNA-Seq datasets. Subsequently, we combined lncRNA annotations from all sources into one non-redundant annotation file. Because there are overlaps between various annotation resources, a procedure was implemented to eliminate redundancy. Among these annotation resources, lncRNAdb contains the most detailed information for ~100 lncRNAs collected from the literature. When transcripts from different sources overlapped, we retained the gene information (gene_id, gene_name etc.) for the loci in the following order of priority: 1) lncRNAdb, 2) GENCODE, 3) RefSeq, 4) Ensembl, or 5) annotated by RNA-Seq experiments. We further combined the resulting lncRNA annotation .gtf with iGenome mm10 gtf annotation and removed any redundancy. This produced a final comprehensive annotation of both non-coding and coding transcripts, which was then used for downstream analysis of RNA-Seq data.

To quantify transcript (both coding and non-coding) expression, we re-ran Cufflink with option –G and supplied the comprehensive .gtf. The multi-read correction and bias correction algorithms in the Cufflinks package were enabled (option: -u -b). Expression level estimation was reported as Fragments per Kilobase of transcript sequence per Million mapped fragment (FPKM) values together with confidence intervals. Transcripts with an FPKM > 0, FPKM_conf_lo (lower bound of confidence interval) > 0, and status = OK were considered as detected with confidence (S2 Table) [[7](#_ENREF_7)].

To facilitate downstream analysis (such as fold change, enrichment analysis, and other procedures), a previously calculated threshold of FPKM = 0.1 was adopted [[1](#_ENREF_1)]. Any FPKM < 0.1 were set to 0.1 for fold enrichment calculations in order to avoid ratio inflation [[12](#_ENREF_12)].

**Potential Functional association of lncRNAs by Gene Set Enrichment Analysis (GSEA)**

We adopted a previously proposed ‘guilt-by-association’ approach to infer potential functions of individual lncRNAs based on their pattern of co-expression with known protein-coding genes [[9](#_ENREF_9)]. We used RNA-Seq data from fifteen types of samples (eight brain cell types and seven non-brain tissues; pericytes were excluded because of their relatively lower purity). RNA-Seq raw data from non-brain tissues were from the Mouse ENCODE project and were downloaded from the Gene Expression Omnibus (GEO accession code GSE36025). All sequence data were analyzed using the same RNA-Seq pipeline described for brain cells. We then computed the Pearson correlations for the expression profile of each protein-coding gene with that of each lncRNA across fifteen types of samples. For each lncRNA, the protein-coding genes were ranked by their correlation coefficient and genes with low correlation (coefficient < |0.2|) were filtered out. This rank of coding genes was then used as input for GSEA, which implements a weighted Kolmogorov-Smirnov (KS) test to identify gene sets that are enriched [[13](#_ENREF_13)]. We performed the test for all lncRNAs against gene sets generated from GO functional terms, Canonical Pathways, and expert-curated gene sets downloaded from the Molecular Signatures Database (MSigDB) [[13](#_ENREF_13)]. Gene sets with less than five genes, or more than 500 genes, were filtered out from the analysis. False discovery rate (FDR) corrected p-values were calculated by permuting gene sets 1000 times.

We then constructed an association matrix between lncRNAs and functional terms. We used a cutoff of FDR < 0. 25 for exploring terms potentially associated with each lncRNA, as recommend by the GSEA manual (S3 Table) [[13](#_ENREF_13)].

**Gene Co-expression Module Analysis**

Weighted gene co-expression network analysis (WGCNA) was conducted to cluster genes into co-expression modules using RNA-Seq data from the purified cell types [[14](#_ENREF_14)]. Briefly, WGCNA identifies modules of co-expressed genes based on a power-transformed Pearson correlation matrix. We first filtered out genes with low expression levels across all samples (e.g., only genes with FPKM > 2 in at least one sample were retained for co-expression analysis). A total of 14,596 genes (including both protein-coding and lncRNA genes) remained for module analysis. Pairwise Pearson correlation coefficients (cor) were first calculated between the expression profiles of all of these genes. Genes were then clustered using hierarchical clustering with complete linkage using (1 – cor) as a distance metric. Clusters with at least 30 members were identified and summarized using eigengene (the first principal component representing the expression pattern of genes in that module). Highly similar modules were merged if the Pearson correlation (cor) between their eigengene values exceeded 0.85. The merging process was iteratively implemented until cor between any pair of modules were under the threshold. A membership value (e.g., cor between a gene and the eigengene value of the module to which it is related) that reflects the relationship of a gene to a module was calculated for every gene and module pair (S5 Table).

For each co-expression module, we then performed functional-term enrichment analysis for modules with > 100 members using DAVID (S4 Table) [[15](#_ENREF_15)].

**DNase I Digital Footprint Data Retrieval and Preprocessing**

DNase I digital footprint data for mouse embryonic stem cell (ESC), whole brain tissue (E14.5 (WBE14) and 8 weeks old adult, (WB8wks)), as well as retina tissue (Retina) were retrieved from the ENCODE project data repository [[16](#_ENREF_16)]. The ENCODE data preprocessing procedure of these datasets can be found at the ENCODE website (<http://genome.ucsc.edu/cgi-bin/hgTrackUi?db=mm9&g=wgEncodeUwDgf>). Briefly, Bowtie 0.1.25 was used to map high-quality read tags back to the mouse genome (mm9). Only unique mapped tags were kept. DNase I hypersensitive zones (HotSpots) were identified using the HotSpot algorithm [[17](#_ENREF_17)]. DNase I hypersensitive sites (DHSs) were identified as signal peaks within hypersensitive zones at a 1.0% false discovery rate. We downloaded the mapped file (.bam) and the peak file (defined DHSs regions) for analysis.

**Identification of Transcription Factor Binding from DNase-DGF Experiments**

In previous literature, the identification of TF binding has been approached in two conceptually distinct ways, either by detecting DNase I footprints first and subsequently matching to previous annotated TF motifs [[18-21](#_ENREF_18)], or by initially detecting TF binding sites genome-wide and subsequently determining whether each site falls within a footprint and has a distinct DNase I cutting profile [[22](#_ENREF_22), [23](#_ENREF_23)]. We chose the latter approach and adopted a previously developed method to detect TF binding in DNase-DGF experiments [[23](#_ENREF_23)].

First, we collected TF motifs from well-known databases including JASPAR [[24](#_ENREF_24)], UniPROBE [[25](#_ENREF_25)], TRANSFAC [[26](#_ENREF_26)], and UW motifs [[27](#_ENREF_27)]. In addition, we surveyed recently published ChIP-Seq datasets for TF motifs that were not available in motif databases, including Ascl1/Mash1 and Olig2, and we *de novo* identified their motifs using the MEME software suite [[28-30](#_ENREF_28)]. Together, we collected 1258 TF motifs from vertebrate species. All motifs were represented in Position Weight Matrix format (PWM). We then scanned the entire mouse reference genome sequence for matches with of PWM of each TF using the genome-wide motif scanning software FIMO (version 4.6.1), setting P < 1e^-5^ as the threshold, based on a previous study [[31](#_ENREF_31), [32](#_ENREF_32)]. Next, we calculated the DNase I cleavage profile around each potential binding site (200-bp window surrounding a given site for both strands) that falls in the DHS region from each DNase-DGF experiment [[23](#_ENREF_23)]. The resulting cleavage profiles for a given TF motif were then combined into a matrix file and were supplied as the input for CENTIPEDE, which is a recently developed algorithm for accurately inferring the binding status of TF motifs. In brief, CENTIPEDE implements an unsupervised Bayesian mixture model to infer which candidate sites for each motif are likely to be bound by a TF, given the experimental data such as DNase I cleavage pattern around a motif. For each potential TF binding site, CENTIPEDE calculates the posterior probability that it is bound by a TF. We subsequently filtered the results. Only sites with posterior probability >= 0.99 were considered as bound by a given TF, which likely corresponds to sites with high TF occupancy[[23](#_ENREF_23)]. The binding site locations calculated for the mm9 genome were mapped to mm10 using the UCSC liftOver utility [[33](#_ENREF_33)].

**Active Regulation of lncRNA by TFs**

To calculate the size of *cis*-regulatory modules, we merged the TF binding sites identified from DNase-DGF experiments, and calculated the number of base pairs that can be bound by TFs and that fall within the promoter regions (defined as 2 kb upstream and 1 kb downstream of the TSS) of TFs, non-TF protein-coding genes, lncRNAs, and randomly selected intergenic regions. The size distributions of *cis*-regulatory modules for each gene category were plotted for comparison. The Mann–Whitney test was calculated to test for significant differences between gene categories.

Gene transcription is regulated in a cell-type and condition-specific manner by TFs. The dynamics of TF binding during development in concert with the dynamics of chromatin accessibility in the regulatory region of lncRNAs can be used to further indicate active regulation [[34](#_ENREF_34)]. We identified dynamic DHSs (△DHS : defined as DHSs that are enriched > threefold under one condition compared to the average of peak signals across all conditions, with peak signal > 10 under the enriched condition to exclude low-signal peaks from the analysis) in the four DNase-DGF experiments.

To identify TF motifs enriched in △DHSs that are activated in different developmental contexts, we performed motif enrichment analysis using sequences of △DHSs that are activated in specific DNase-DGF experiments. The analysis was performed using HOMER software suite, which implements a binomial distribution for the calculation of p-values for the significance of enrichment [[35](#_ENREF_35)].

**Identification of Potential Transcription Factor Binding Sites Using ENCODE Motifs and Promoters**

In addition to DNase-DGF data, we performed motif searches in the promoter regions annotated by the Mouse ENCODE project [[36](#_ENREF_36), [37](#_ENREF_37)]. We downloaded 2065 ENCODE motifs (<http://compbio.mit.edu/encode-motifs>) and combined them with 12 motifs identified *de novo* from the ChIP-Seq data for Ascl1/Mash1 and Olig2, as previously described. A total of 618 unique transcription factors with 2077 motifs were used to scan the proximal regions of lncRNA sequences using FIMO using default parameters. The total number of predicted transcription factor binding sites (TFBS) for selected lncRNAs was 50,149. To further filter the predicted TFBS within lncRNA upstream regulatory regions (defined as 4 kb upstream and 1 kb downstream of the TSS), we used a list of 82,853 putative promoters annotated by the Mouse ENCODE project [[36](#_ENREF_36)]. Promoter sequences were first converted into mm10 using the UCSC liftOver utility and then the intersection between the predicted TFBS and promoter regions was calculated [[33](#_ENREF_33)]. To analyze correlations between the expression of TFs and the target lncRNA proximal regions containing the corresponding TF motifs, we computed the Pearson correlation coefficient between the expression of the TFs and the target lncRNAs. A heatmap representing the correlation of expression between TFs and target lncRNAs was constructed (S4 Fig.).

**Differential Expression Analysis of RNA-Seq Data**

To identify protein-coding and lncRNA genes that are regulated during the generation of OPCs, we performed differential expression analysis using NSC and OPC RNA-Seq data. In brief, the htseq-count script distributed with the HTSeq Python package (<http://www-huber.embl.de/users/anders/HTSeq/doc/count.html>) was used for calculating raw read counts for each gene feature as input for DESeq [[38](#_ENREF_38)]. DE genes were called using the DESeq package according to the developer’s instructions (<http://bioconductor.org/packages/2.13/bioc/html/DESeq.html>) [[39](#_ENREF_39)]. Genes with FDR < 0.05 were considered as significant differentially expressed genes.

Functional term enrichment analysis was performed using DAVID [[15](#_ENREF_15)].

**Comparative Genome Analysis**

The UCSC genome browser was used for comparative genome analysis [[33](#_ENREF_33)]. Multiz Align track, Placental Conservation track, and RepeatMask track were enabled to inspect insertions and PhyliP conservation scores, and to search for evidence of TEs.

**Oligonucleotide sequences used in the study**

All primer sequences used for qPCR to analyze oligodendrocyte differentiation are listed. Upon induction of oligodendrocyte differentiation, GAPDH, myelin basic protein (MBP), CNP, and proteolipid peptide (PLP) mRNA expression were measured by qRT-PCR. Primers used for *lnc-OPC* expression analysis are also included in the following table. GAPDH expression was analyzed and used as an internal control to calculate the relative expression values of other genes. Primers used for OLIG2 ChIP-qPCR are also listed.

**Table 1. Oligonucleotide sequences used for qRT-PCR experiments**

| GAPDH | qRT-PCR Forward primer | ATGACATCAAGAAGGTGGTG |
| --- | --- | --- |
| GAPDH | qRT-PCR Reverse primer | CATACCAGGAAATGAGCTTG |
| CNP-1 | qRT-PCR Forward primer | TACTTCGGCTGGTTCCTGAC |
| CNP-1 | qRT-PCR Reverse primer | GCCTTCCCGTAGTCACAAAA |
| PLP | qRT-PCR Forward primer | AGCAAAGTCAGCCGCAAAAC |
| PLP | qRT-PCR Reverse primer | CCAGGGAAGCAAAGGGGG |
| MBP | qRT-PCR Forward primer | CTATAAATCGGCTCACAAGG |
| MBP | qRT-PCR Reverse primer | AGGCGGTTATATTAAGAAGC |
| *Lnc-OPC* | qRT-PCR Forward primer | ACTAACCTTCTGTTCTTCTACCTT |
| *Lnc-OPC* | qRT-PCR Reverse primer | ATTTAGACTCAGCACACACTTTC |

**Table 2. Oligonucleotide sequences used for shRNA experiments**

| luciferase control | shRNA target sequence | ATGACATCAAGAAGGTGGTG |
| --- | --- | --- |
| sh-lnc-OPC2 | shRNA target sequence | GAACGTCACTAACCTTCTGTT |
| sh-lnc-OPC3 | shRNA target sequence | GTGCAAGCAGGATACCTAAAG |
| Control(sh-luc) | sense sequence | CCGGGGCTATGAAGAGATAC  GCCCTGCAGGGCGTATCTCTT  CATAGCCTTTTTG |
| Control(sh-luc) | antisense sequence | AATTCAAAAAGCTATGAAGA  GATACGCCCTGCAGGGCGTA  TCTCTTCATAGCC |
| sh-lnc-OPC2 | sense sequence | CCGGGAACGTCACTAACCTT  CTGTTCTGCAGAACAGAAGG  TTAGTGACGTTCTTTTTG |
| sh-lnc-OPC2 | antisense sequence | AATTCAAAAAGAACGTCACT  AACCTTCTGTTCTGCAGAAC  AGAAGGTTAGTGACGTTC |
| sh-lnc-OPC3 | sense sequence | CCGGGTGCAAGCAGGATAC  CTAAAGCTGCAGCTTTAGGT  ATCCTGCTTGCACTTTTTG |
| sh-lnc-OPC3 | antisense sequence | AATTCAAAAAGTGCAAGCA  GGATACCTAAAGCTGCAGC  TTTAGGTATCCTGCTTGCAC |

**Table 3. Oligonucleotide sequences used for OLIG2 ChIP-qPCR experiments**

| Primer1 | qPCR Forward primer | ATCGCTACACGGTTGCTAT |
| --- | --- | --- |
| Primer1 | qPCR Reverse primer | CACTGTCACTGCCCTTCT |
| Primer2 | qPCR Forward primer | AACAGTCGTGTCTGAACCA |
| Primer2 | qPCR Reverse primer | TGAATTAACCAGCAAGCCATT |
| Negative | qPCR Forward primer | CCTGGGAAAGTGTGACAAATG |
| Negative | qPCR Reverse primer | TGGTTGGAGCCTCTTCTTG |

**Table 4. Oligonucleotide sequences used for constructing luciferase reporter plasmids**

| A1 construct PCR primer | Forward primer | CGGGGTACCCTTGTGCCAAGCACTTTCCC |
| --- | --- | --- |
| A1 construct PCR primer | Reverse primer | CCGCTCGAGTGGCTTGCTGGTTAATTCATCG |
| A2 construct PCR primer | Forward primer | CGGGGTACCGGCTTGTCTCAGCTCATGGT |
| A2 construct PCR primer | Reverse primer | CCGCTCGAGGGCTTGCTGGTTAATTCATCG |

1. Zhang Y, Chen K, Sloan Sa, Bennett ML, Scholze AR, O'Keeffe S, et al. An RNA-Sequencing Transcriptome and Splicing Database of Glia, Neurons, and Vascular Cells of the Cerebral Cortex. J Neurosci. 2014;34:11929–11947. doi: 10.1523/JNEUROSCI.1860-14.2014. PMID: 25186741.

2. Chen K, Deng S, Lu H, Zheng Y, Yang G, Kim D, et al. RNA-Seq Characterization of Spinal Cord Injury Transcriptome in Acute/Subacute Phases: A Resource for Understanding the Pathology at the Systems Level. PloS one. 2013;8:e72567. doi: 10.1371/journal.pone.0072567. PMID: 23951329.

3. Trapnell C, Pachter L, Salzberg SL. TopHat: discovering splice junctions with RNA-Seq. Bioinformatics. 2009;25:1105–1111. doi: 10.1093/bioinformatics/btp120. PMID: 19289445.

4. Langmead B, Trapnell C, Pop M, Salzberg SL. Ultrafast and memory-efficient alignment of short DNA sequences to the human genome. Genome Biol. 2009;10(3):R25. Epub 2009/03/06. doi:10.1186/gb-2009-10-3-r25. PMID: 19261174.

5. Trapnell C, Roberts A, Goff L, Pertea G, Kim D, Kelley DR, et al. Differential gene and transcript expression analysis of RNA-seq experiments with TopHat and Cufflinks. Nat Protoc. 2012;7:562–578. doi: 10.1038/nprot.2012.016. PMID: 22383036.

6. Necsulea A, Soumillon M, Warnefors M, Liechti A, Daish T, Zeller U, et al. The evolution of lncRNA repertoires and expression patterns in tetrapods. Nature. 2014;505:635–640. doi:10.1038/nature12943. PMID: 24463510.

7. Ramos AD, Diaz A, Nellore A, Delgado RN, Park K-Y, Gonzales-Roybal G, et al. Integration of genome-wide approaches identifies lncRNAs of adult neural stem cells and their progeny in vivo. Cell stem cell. 2013;12:616–628. doi: 10.1016/j.stem.2013.03.003. PMID: 23583100.

8. Wang L, Park HJ, Dasari S, Wang S, Kocher JP, Li W. CPAT: Coding-Potential Assessment Tool using an alignment-free logistic regression model. Nucleic Acids Res. 2013;41(6):e74. Epub 2013/01/22. doi:10.1093/nar/gkt006. PMID: 23335781.

9. Guttman M, Amit I, Garber M, French C, Lin MF, Feldser D, et al. Chromatin signature reveals over a thousand highly conserved large non-coding RNAs in mammals. Nature. 2009;458:223–227. doi:10.1038/nature07672. PMID: 19182780.

10. Guttman M, Garber M, Levin JZ, Donaghey J, Robinson J, Adiconis X, et al. Ab initio reconstruction of cell type-specific transcriptomes in mouse reveals the conserved multi-exonic structure of lincRNAs. Nat Biotechnol. 2010;28:503–510. doi: 10.1038/nbt.1633. PMID: 20436462.

11. Belgard TG, Marques AC, Oliver PL, Abaan HO, Sirey TM, Hoerder-Suabedissen A, et al. A transcriptomic atlas of mouse neocortical layers. Neuron. 2011;71:605–616. doi:10.1016/j.neuron.2011.06.039. PMID: 21867878.

12. Quackenbush J. Microarray data normalization and transformation. Nat Genet. 2002;32 Suppl:496–501. Epub 2002/11/28. doi: 10.1038/ng1032. PMID: 12454644.

13. Subramanian A, Tamayo P, Mootha VK, Mukherjee S, Ebert BL, Gillette MA, et al. Gene set enrichment analysis: a knowledge-based approach for interpreting genome-wide expression profiles. Proc Natl Acad Sci U S A. 2005;102(43):15545–15550. Epub 2005/10/04. doi:10.1073/pnas.0506580102. PMID: 16199517.

14. Oldham MC, Konopka G, Iwamoto K, Langfelder P, Kato T, Horvath S, et al. Functional organization of the transcriptome in human brain. Nat Neurosci. 2008;11:1271–1282. doi:10.1038/nn.2207. PMID: 18849986.

15. Huang da W, Sherman BT, Lempicki RA. Systematic and integrative analysis of large gene lists using DAVID bioinformatics resources. Nat Protoc. 2009;4(1):44–57. Epub 2009/01/10. doi:10.1038/nprot.2008.211. PMID: 19131956.

16. Encode T, Consortium P. A user's guide to the encyclopedia of DNA elements (ENCODE). PLoS Biol. 2011;9:e1001046. doi: 10.1371/journal.pbio.1001046. PMID: 21526222.

17. Sabo PJ, Hawrylycz M, Wallace JC, Humbert R, Yu M, Shafer A, et al. Discovery of functional noncoding elements by digital analysis of chromatin structure. Proc Natl Acad Sci U S A. 2004;101:16837–16842. doi: 10.1073/pnas.0407387101. PMID: 15550541.

18. Trompouki E, Bowman TV, Lawton LN, Fan ZP, Wu DC, Dibiase A, et al. Lineage regulators direct BMP and Wnt pathways to cell-specific programs during differentiation and regeneration. Cell. 2011;147:577–589. doi: 10.1016/j.cell.2011.09.044. PMID: 22036566.

19. Young RA. Control of the embryonic stem cell state. Cell. 2011;144(6):940–954. Epub 2011/03/19. doi: 10.1016/j.cell.2011.01.032. PMID: 21414485.

20. Davidson EH. Emerging properties of animal gene regulatory networks. Nature. 2010;468:911–920. doi: 10.1038/nature09645. PMID: 21164479.

21. Birney E, Stamatoyannopoulos Ja, Dutta A, Guigó R, Gingeras TR, Margulies EH, et al. Identification and analysis of functional elements in 1% of the human genome by the ENCODE pilot project. Nature. 2007;447:799–816. doi: 10.1038/nature05874. PMID: 17571346.

22. Guo Y, Mahony S, Gifford DK. High Resolution Genome Wide Binding Event Finding and Motif Discovery Reveals Transcription Factor Spatial Binding Constraints. PLoS Comput Biol. 2012;8. doi: 10.1371/journal.pcbi.1002638. PMID: 22912568.

23. Pique-Regi R, Degner JF, Pai Aa, Gaffney DJ, Gilad Y, Pritchard JK. Accurate inference of transcription factor binding from DNA sequence and chromatin accessibility data. Genome Res. 2011;21:447–455. doi: 10.1101/gr.112623.110. PMID: 21106904.

24. Sandelin A, Alkema W, Engström P, Wasserman WW, Lenhard B. JASPAR: an open-access database for eukaryotic transcription factor binding profiles. Nucleic Acids Res. 2004;32:D91–94. doi: 10.1093/nar/gkh012. PMID: 14681366.

25. Newburger DE, Bulyk ML. UniPROBE: an online database of protein binding microarray data on protein-DNA interactions. Nucleic Acids Res. 2009;37:D77–82. doi: 10.1093/nar/gkn660. PMID: 18842628.

26. Matys V, Fricke E, Geffers R, Gossling E, Haubrock M, Hehl R, et al. TRANSFAC: transcriptional regulation, from patterns to profiles. Nucleic Acids Res. 2003;31(1):374–378. Epub 2003/01/10. PMID: 12520026.

27. Neph S, Vierstra J, Stergachis AB, Reynolds AP, Haugen E, Vernot B, et al. An expansive human regulatory lexicon encoded in transcription factor footprints. Nature. 2012;489:83–90. doi:10.1038/nature11212. PMID: 22955618.

28. Bailey TL, Boden M, Buske FA, Frith M, Grant CE, Clementi L, et al. MEME Suite: Tools for motif discovery and searching. Nucleic Acids Res. 2009;37. doi: 10.1093/nar/gkp335. PMID: 19458158.

29. Wapinski OL, Vierbuchen T, Qu K, Lee QY, Chanda S, Fuentes DR, et al. Hierarchical mechanisms for direct reprogramming of fibroblasts to neurons. Cell. 2013;155(3):621–635. Epub 2013/11/19. doi:10.1016/j.cell.2013.09.028. PMID: 24243019.

30. Mazzoni EO, Mahony S, Iacovino M, Morrison CA, Mountoufaris G, Closser M, et al. Embryonic stem cell-based mapping of developmental transcriptional programs. Nat Methods. 2011;8(12):1056–1058. Epub 2011/11/15. doi: 10.1038/nmeth.1775. PMID: 22081127.

31. Grant CE, Bailey TL, Noble WS. FIMO: scanning for occurrences of a given motif. Bioinformatics. 2011;27:1017–1018. doi: 10.1093/bioinformatics/btr064. PMID: 21330290.

32. Neph S, Stergachis AB, Reynolds A, Sandstrom R, Borenstein E, Stamatoyannopoulos JA. Circuitry and dynamics of human transcription factor regulatory networks. Cell. 2012;150(6):1274–1286. Epub 2012/09/11. doi: 10.1016/j.cell.2012.04.040. PMID: 22959076.

33. Meyer LR, Zweig AS, Hinrichs AS, Karolchik D, Kuhn RM, Wong M, et al. The UCSC Genome Browser database: extensions and updates 2013. Nucleic Acids Res. 2013;41(Database issue):D64–69. Epub 2012/11/17. doi: 10.1093/nar/gks1048. PMID: 23155063.

34. He HH, Meyer CA, Chen MW, Jordan VC, Brown M, Liu XS. Differential DNase I hypersensitivity reveals factor-dependent chromatin dynamics. Genome Res. 2012;22(6):1015–1025. Epub 2012/04/18. doi: 10.1101/gr.133280.111. PMID: 22508765.

35. Heinz S, Benner C, Spann N, Bertolino E, Lin YC, Laslo P, et al. Simple combinations of lineage-determining transcription factors prime cis-regulatory elements required for macrophage and B cell identities. Mol Cell. 2010;38(4):576–589. Epub 2010/06/02. doi: 10.1016/j.molcel.2010.05.004. PMID: 20513432.

36. Yue F, Cheng Y, Breschi A, Vierstra J, Wu W, Ryba T, et al. A comparative encyclopedia of DNA elements in the mouse genome. Nature. 2014;515(7527):355–364. Epub 2014/11/21. doi:10.1038/nature13992. PMID: 25409824.

37. Kheradpour P, Kellis M. Systematic discovery and characterization of regulatory motifs in ENCODE TF binding experiments. Nucleic Acids Res. 2014;42(5):2976–2987. Epub 2013/12/18. doi:10.1093/nar/gkt1249. PMID: 24335146.

38. Anders S, Pyl PT, Huber W. HTSeq-a Python framework to work with high-throughput sequencing data. Bioinformatics. 2014. Epub 2014/09/28. doi:10.1093/bioinformatics/btu638. PMID: 25260700.

39. Anders S, Huber W. Differential expression analysis for sequence count data. Genome Biol. 2010;11(10):R106. Epub 2010/10/29. doi: 10.1186/gb-2010-11-10-r106. PMID: 20979621.
